# β2-Syntrophin Is a Cdk5 Substrate That Restrains the Motility of Insulin Secretory Granules

**DOI:** 10.1371/journal.pone.0012929

**Published:** 2010-09-23

**Authors:** Sandra Schubert, Klaus-Peter Knoch, Joke Ouwendijk, Shabaz Mohammed, Yury Bodrov, Melanie Jäger, Anke Altkrüger, Carolin Wegbrod, Marvin E. Adams, Yong Kim, Stanley C. Froehner, Ole N. Jensen, Yannis Kalaidzidis, Michele Solimena

**Affiliations:** 1 Molecular Diabetology, Paul Langerhans Institute Dresden, Uniklinikum Carl Gustav Carus at Dresden University of Technology, Dresden, Germany; 2 Max Planck Institute of Molecular Cell Biology and Genetics, Dresden, Germany; 3 Department of Biochemistry and Molecular Biology, University of Southern Denmark, Odense, Denmark; 4 Department of Physiology and Biophysics, University of Washington, Seattle, Washington, United States of America; 5 Laboratory of Molecular and Cellular Neuroscience, The Rockefeller University, New York, New York, United States of America; 6 A. N. Belozersky Institute of Physico-Chemical Biology, Moscow State University, Moscow, Russia; University of Bremen, Germany

## Abstract

The molecular basis for the interaction of insulin granules with the cortical cytoskeleton of pancreatic β-cells remains unknown. We have proposed that binding of the granule protein ICA512 to the PDZ domain of β2-syntrophin anchors granules to actin filaments and that the phosphorylation/dephosphorylation of β2-syntrophin regulates this association. Here we tested this hypothesis by analyzing INS-1 cells expressing GFP-β2-syntrophin through the combined use of biochemical approaches, imaging studies by confocal and total internal reflection fluorescence microscopy as well as electron microscopy. Our results support the notion that β2-syntrophin restrains the mobility of cortical granules in insulinoma INS-1 cells, thereby reducing insulin secretion and increasing insulin stores in resting cells, while increasing insulin release upon stimulation. Using mass spectrometry, *in vitro* phosphorylation assays and β2-syntrophin phosphomutants we found that phosphorylation of β2-syntrophin on S75 near the PDZ domain decreases its binding to ICA512 and correlates with increased granule motility, while phosphorylation of S90 has opposite effects. We further show that Cdk5, which regulates insulin secretion, phosphorylates S75. These findings provide mechanistic insight into how stimulation displaces insulin granules from cortical actin, thus promoting their motility and exocytosis.

## Introduction

Glucose-stimulated trafficking of secretory granules of pancreatic β-cells is required for sustained insulin secretion [1]. Increasing evidence points to a dual role for the cortical actin cytoskeleton in regulating the availability of neurosecretory granules, including insulin granules, for exocytosis [2], [3]. Actin filaments support the short-range movements of granules [4]–[6], but they can also restrict the mobility of vesicles and their access to the plasma membrane [7]–[9]. Little is known, however, about how granules interact with the cortical cytoskeleton.

The catalytically inactive receptor protein tyrosine phosphatase N, also known as islet cell autoantigen 512 (ICA512) or IA-2 [10]–[12], is an intrinsic membrane protein of secretory granules [13] which may link granules to actin filaments [14]. Specifically, its cytoplasmic tail binds to the N-terminal PSD95/Dlg/ZO-1 (PDZ) domain of β2-syntrophin [15], [16], which in turn interacts with F-actin through utrophin [17], [18]. β2-syntrophin is the prevalent β-cell isoform of a highly conserved family of 58 kDa modular adapter proteins, termed the Syntrophins [19], [20]. The five syntrophin isoforms (α1, β1, β2, γ1, γ2) have a common domain organization. Their N-terminal region includes a PDZ domain embedded within a pleckstrin homology (PH1) domain. The C-terminal half contains a second PH (PH2) domain and the highly conserved syntrophin unique (SU) domain [21]. The PH2 and SU domains are both required for binding dystrophin and the dystrophin-related proteins utrophin and dystrobrevin [22], [23], which in turn bind actin [24]. Syntrophins are part of the dystrophin associated protein complex (DAPC), which links the actin cytoskeleton with the extracellular matrix (ECM) [25]. The DAPC is also implicated in transmembrane signaling [17]. α1- and β2-syntrophins, in particular, bind to neuronal nitric oxide synthase (nNOS) [26], [27], syntrophin-associated serine/threonine (SAST) kinase [28], stress-activated protein kinase 3 and voltage-gated Na+ channel (VGSC) [29], [30] via the PDZ domain. Evidence that syntrophins are implicated in membrane traffic is supported by the finding that knockout of α1-syntrophin alters the perivascular localization of aquaporin-4-containing vesicles [31]–[33].

Previous studies suggested that β2-syntrophin is phosphorylated at multiple sites in rat insulinoma INS-1 cells and that dephosphorylation following stimulation of insulin secretion weakens its binding to ICA512 and the anchoring of insulin granules to microfilaments [16]. Here we tested this hypothesis by performing extensive imaging and biochemical studies in INS-1 cells.

## Results

### GFP-β2-syntrophin resembles endogenous β2-syntrophin

Since primary beta cells are not well suited for in-depth mechanistic studies, INS-1 cells were chosen as an in vitro model to gain further insight into the phosphorylation and function of β2-syntrophin. The expression pattern of β2-syntrophin in INS-1 cells resembled closely that of rat and mouse pancreatic islets (Fig. S1A).

**Figure 1:**
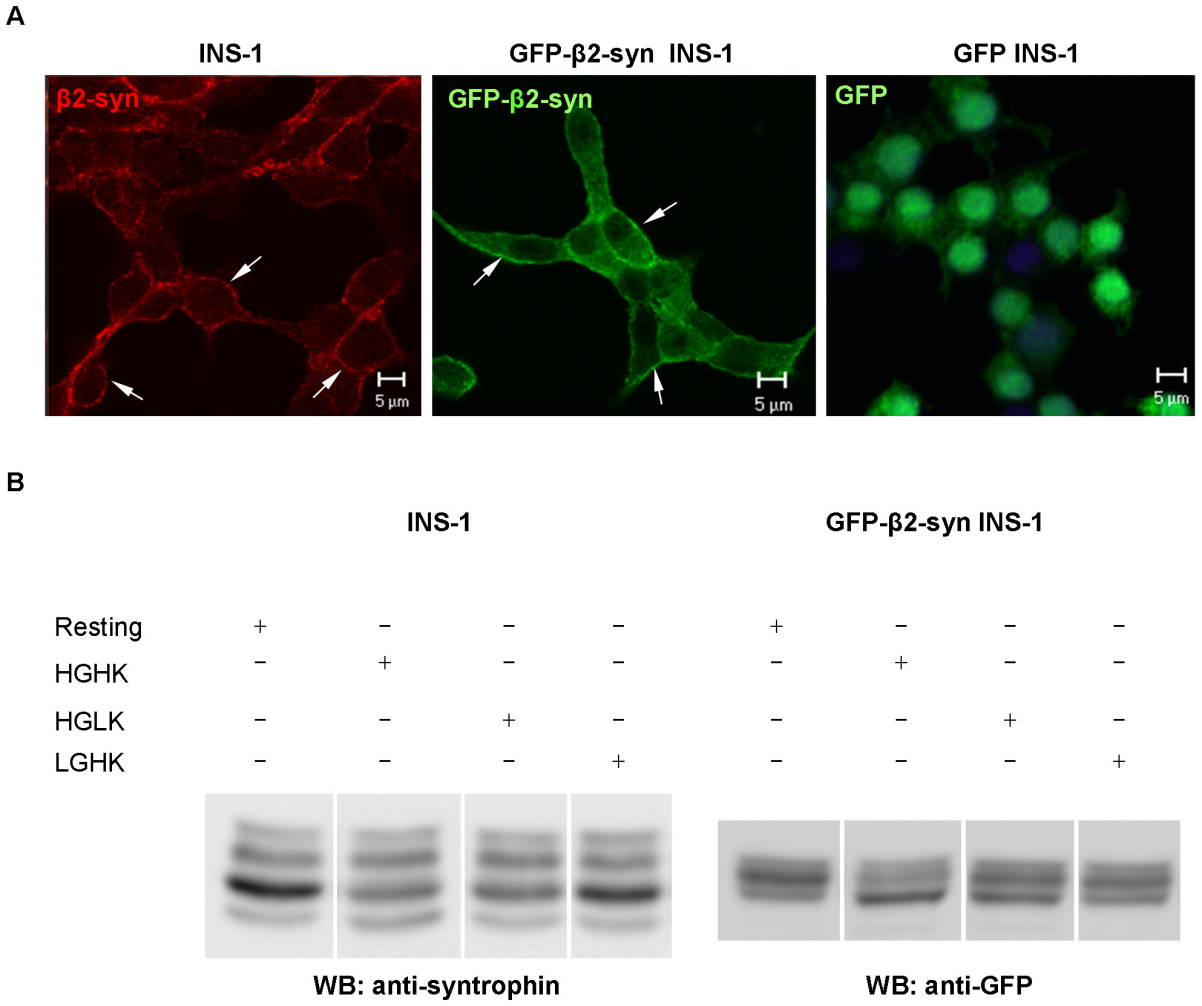
Expression, localization and stimulus-dependent regulation of β2-syntrophin. **A**) Confocal microscopy for β2-syntrophin in INS-1 cells and for GFP-β2-syntrophin or GFP in transfected INS-1 cells. β2-syntrophin was detected with the pan-syntrophin antibody 1351. White arrows indicate the enrichment of β2-syntrophin at the cell periphery. Bars: 5 µm. **B**) Western blots (WB) with anti-syntrophin or anti-GFP antibodies on extracts of INS-1 cells (left panel) or GFP-β2-syntrophin INS-1 cells (right panel). Cells were previously kept at rest (0 mM glucose, 5 mM KCl) or stimulated with high (25 mM) glucose and low (5 mM) KCl (HGLK), low (0 mM) glucose and high (55 mM) KCl (LGHK) or high glucose and high KCl (HGHK).

Previous studies suggested that sustained stimulation of INS-1 cells with 25 mM glucose (high glucose, HG) plus 55 mM KCl (high potassium, HK) altered the electrophoretic pattern of β2-syntrophin due to changes in the ratio among different phosphorylated species [16]. However, the low expression of β2-syntrophin precluded a detailed analysis of its phosphorylation. Therefore we generated INS-1 cell clones that stably expressed β2-syntrophin N-terminally fused with GFP (GFP-β2-syntrophin). As endogenous β2-syntrophin, GFP-β2-syntrophin was restricted to the cell cortex (Fig. 1A). β2-syntrophin and GFP-β2-syntrophin displayed a similar complex electrophoretic pattern (Fig. 1B). Following stimulation with HG, either alone or together with HK, the signal of the most prominent species in resting cells was reduced, while the fastest migrating species was enhanced, conceivably due to dephosphorylation (Fig. 1B). Stimulation with HG also slightly increased a slower migrating species, presumably due to phosphorylation. This species was also enhanced in rat islets stimulated with HG, alone or together with HK (Fig. S1B). Stimulation with HK alone induced less prominent changes. Given its resemblance to the endogenous protein, GFP-β2-syntrophin was exploited to study the phosphorylation and function of β2-syntrophin in INS-1 cells.

### β2-syntrophin modulates insulin secretion

**Figure 2:**
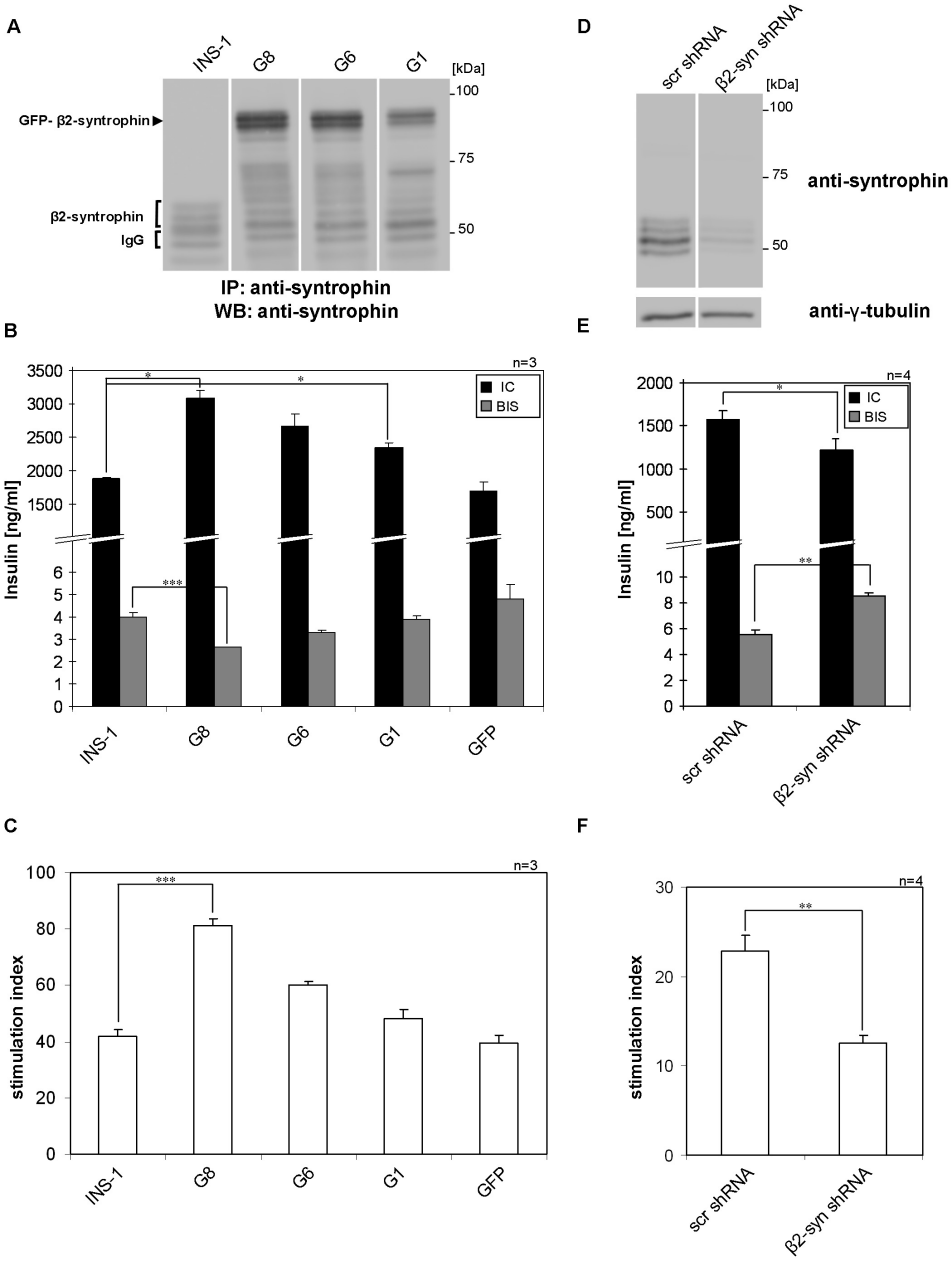
Insulin content and secretion of INS-1 cells in relation to β2-syntrophin expression levels. **A**) Immunoblots with the anti-syntrophin antibody on immunoprecipitates obtained with the same antibody from extracts of INS-1 cells and three INS-1 cell clones (G1, G6 and G8) stably transfected with GFP-β2-syntrophin. **B**) Insulin content (IC) and basal insulin secretion (BIS) of INS-1 cells, GFP-β2-syntrophin INS-1 cell clones G1, G6, G8 and INS-1 cells stably transfected with GFP. **C**) Insulin secretion Stimulation Index (SI) of INS-1 cells, GFP-β2-syntrophin INS-1 cell clones G1, G6, G8 and GFP INS-1 cells. **D**) Immunoblots with anti-syntrophin and anti-γ-tubulin antibodies on extracts of INS-1 cells transfected with a scrambled shRNA (scr shRNA) or an shRNA for the knockdown of β2-syntrophin (β2-syn shRNA). **E**) IC and BIS of INS-1 cells transfected with β2-syn or scr shRNAs. **F**) SI of INS-1 cells transfected with β2-syn or scr shRNAs. n =  number of independent experiments; *, p = 0.05; **, p = 0.01; ***, p = 0.005. p-values in B and C are relative to INS-1 cells, while in E and F they are relative to INS-1 cells transfected with scr shRNA.

First, we analyzed if GFP-β2-syntrophin affects insulin secretion. To this aim we selected three INS-1 cell clones (G1, G6, G8) in which GFP-β2-syntrophin was stably expressed 1, 1.8 or 2.5 fold more than endogenous β2-syntrophin (not considering the presumably GFP-β2-syntrophin-derived fragment of ∼70 kDa) (Fig. 2A). These clones were compared to wild type and GFP-expressing INS-1 cells for insulin content and release. The basal insulin secretion was decreased, while both insulin content and the HGHK insulin secretion stimulation index were increased accordingly to the levels of GFP-β2-syntrophin (Fig. 2B–C and Table S1A). The expression of GFP alone did not affect any of these parameters relative to wild type INS-1 cells. Conversely, ∼70% knockdown of β2-syntrophin expression by RNA interference (RNAi) significantly increased basal insulin secretion compared to INS-1 cells transfected with a scrambled hairpin for RNAi, while the levels of cellular insulin and the stimulation index were significantly decreased (Fig. 2D–F and Table S1B). These effects were specific as they were rescued by the overexpression of GFP-β2-syntrophin (Table S1B and Fig. S2). An explanation for these findings is that restraint of granules by β2-syntrophin reduces their basal exocytosis, thereby increasing insulin stores and their release upon stimulation.

### β2-syntrophin slows the mobility of cortical granules

**Figure 3:**
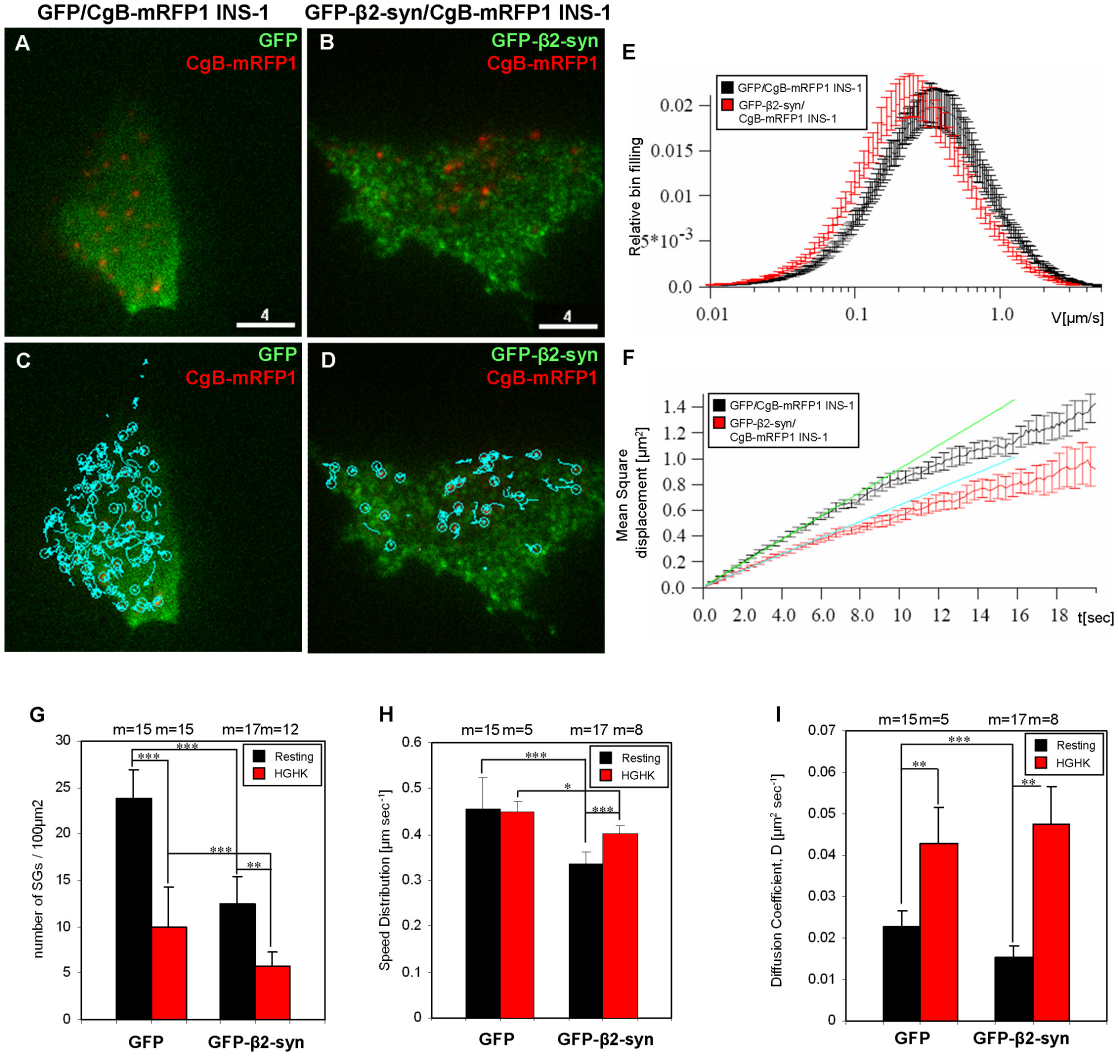
Granule density and mobility in GFP-β2-syntrophin INS-1 cells. **A–D**) Frames from TIRFM movies tracking the motion of CgB-mRFP1^+^ granules (red) in resting INS-1 cells expressing GFP (GFP/CgB-mRFP1 INS-1) (**A**, **C**) or GFP-β2-syntrophin (GFP-β2-syn/CgB-mRFP1 INS-1) (**B**, **D**). In **C** and **D** each blue line shows the motion of an individual CgB-mRFP1^+^ granule tracked for 40 sec. Scale bars: 4 µm. **E**, **F**) Mean speed velocity (**E**) and square displacement (**F**) of CgB-mRFP1^+^ granules in resting INS-1 cells expressing GFP (black) or GFP-β2-syntrophin (red). In **F** the green and blue lines show the weighted least square fit of the initial (first 4 sec) mean square displacement points. The mean square displacement slope of linear dependency is K = 4D, where D is the diffusion coefficient in µm^2^ sec^−1^. **G**) Density of granules expressed as the number of granules/100 µm^2^ in resting (black bars) and stimulated (red bars) GFP/and GFP-β2-syntrophin/CgB-mRFP1 INS-1 cells. **H**, **I**) Mean speed velocities (**H**) and diffusion coefficients (**I**) of CgB-mRFP1^+^ granules in resting (black bars) or HGHK-stimulated (red bars) INS-1 cells expressing GFP or GFP-β2-syntrophin. m =  number of movies examined for each condition from four independent experiments.

The cortical actin cytoskeleton influences the recruitment of granules for exocytosis by preventing their access to the plasma membrane. Major changes in the assembly of the cortical cytoskeleton are unlikely to account for the effect of β2-syntrophin on insulin secretion (Fig. S3A) since the staining pattern of F-actin in GFP-β2-syntrophin and GFP INS-1 cells were comparable. If β2-syntrophin restrains cortical granules, its overexpression should reduce their mobility. To test this hypothesis we used total internal reflection fluorescence microscopy (TIRFM) to analyze granule mobility and density near (≤200 nm) the plasma membrane [34], [35]. Granules were visualized after stable transfection of INS-1 cells with the granule cargo chromogranin B fused to monomeric Red Fluorescent Protein 1 (CgB-mRFP1). Co-localization of CgB-mRFP1 with insulin granules was verified by confocal microscopy (Fig. S3B). Granule motility was automatically tracked and quantified with the MotionTracking/Kalaimoscope software (Fig. 3A–D and Movies S1–S2). In resting and HGHK-stimulated CgB-mRFP1 INS-1 cells transiently co-expressing GFP-β2-syntrophin the density of cortical granules was reduced to 42% and 46% relative to GFP/CgB-mRFP1 INS-1 cells, respectively (Fig. 3G and Table S2A). However, stimulation with HGHK reduced the density of cortical granules by ∼1.8 fold in both GFP-β2-syntrophin/CgB-mRFP1 and GFP/CgB-mRFP1 INS-1 cells; consistent with the hypothesis that β2-syntrophin regulates the recruitment of granules to the plasma membrane rather than a late step in exocytosis. Accordingly, the mean speed of granules in resting GFP-β2-syntrophin/CgB-mRFP1 INS-1 cells was 74% compared to GFP/CgB-mRFP1 INS-1 cells (Fig. 3E and H and Table S2B). HGHK stimulation increased granule mobility in GFP-β2-syntrophin/CgB-mRFP1 INS-1 cells by 120%, while it did not changed it in GFP/CgB-mRFP1 INS-1 cells (Fig. 3H and Table S2B).

Further insights into the impact of β2-syntrophin on the kinetics of granules were obtained by analyzing their average mean square displacement. An almost linear dependency of the mean-square displacement is characteristic of vesicles whose motion is not constrained [36]. This pattern arises from frequent and random changes in the direction of granule movements. Here, the mean square displacement (K) is indicated as diffusion coefficient (D) and K = 4D at a slope of linear dependency (Fig. 3F and I, Table S2C). In resting GFP-β2-syntrophin/CgB-mRFP1 INS-1 cells the granule diffusion coefficient was reduced to 65% compared to GFP/CgB-mRFP1 INS-1 cells. This behavior is characteristic of vesicles with a stronger interaction with the cytoskeleton and corroborates the hypothesis that β2-syntrophin restrains granule mobility. Relaxation of this constraint upon stimulation could then allow granules to move more freely. Accordingly, after stimulation the granule diffusion coefficient increased to comparable levels in GFP-β2-syntrophin/CgB-mRFP1 and GFP/CgB-mRFP1 INS-1 cells (Fig. 3I and Table S2C).

We further compared the appearance of granules in control and GFP-β2-syntrophin INS-1 cells by electron microscopy (Fig. S4). In GFP-β2-syntrophin INS-1 cells, the number of granules/cell was increased ∼1.8 fold compared to control INS-1 cells (26.9±5.8 vs. 14.8±2.6, p = 0.05), while their size was ∼1.3 fold larger (84540±5696 nm2 vs. 67172±4584 nm2, p = 0.02). These data are consistent with the greater insulin content measured in GFP-β2-syntrophin INS-1 cells. We also observed that a minor fraction of granules in resting GFP-β2-syntrophin INS-1 cells (1.51%) and INS-1 cells (1.63%) were pear-shaped rather than spheroidal, being their major/minor diameter ratio >1.9 (Fig. S4A). Oblong granules were also observed in specimen that had been either fixed by high pressure freezing or processed for cryoimmunolabeling (Fig. S4A). Intriguingly, in GFP-β2-syntrophin INS-1 cells kept in culture media, which contains 11 mM glucose and sub-maximally stimulates insulin secretion (Fig. S4C), the percentage of deformed granules increased to 6% compared to 1.8% (p = 0.02) in control INS-1 cells (Fig. S4B and Table S3). Whether this deformed morphology reflects granule transport remains to be determined.

### β2-syntrophin is phosphorylated on four serine sites

**Figure 4:**
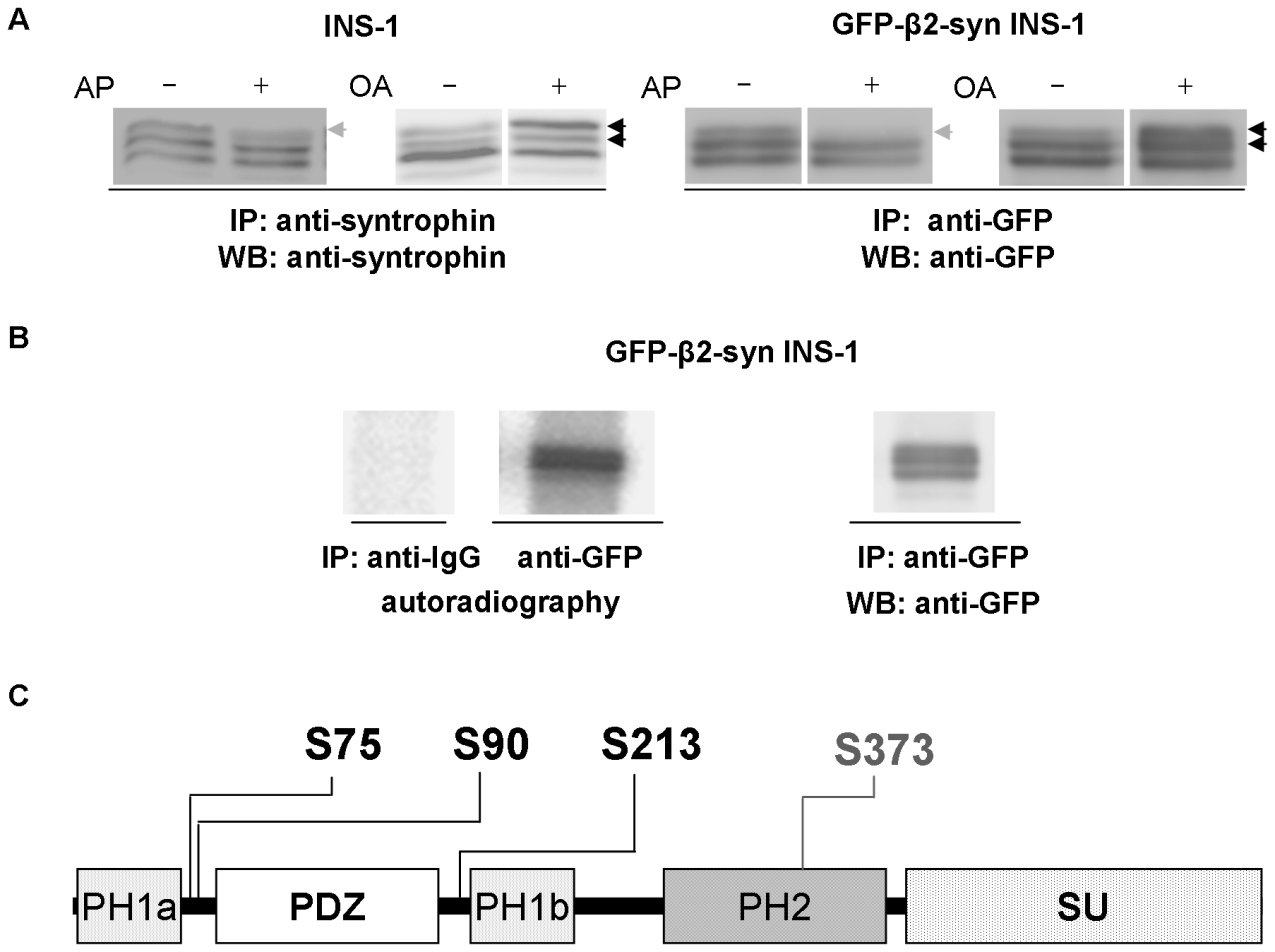
Phosphorylation/dephosporylation of β2-syntrophin. **A**) Immunoblots with the anti-syntrophin or anti-GFP antibodies following immunoprecipitations with the same antibodies from extracts of INS-1 and GFP-β2-syntrophin INS-1 cells, respectively. Alkaline phosphatase (AP) was added to the immunoprecipitates, while okadaic acid (OA) was added before cell extraction. β2-syntrophin species the levels of which are reduced upon AP treatment are marked with a gray arrow, while black arrows indicated species increased upon OA incubation. **B**) The two panels on the left show the autoradiographies of ^32^P-GFP-β2-syntrophin immunoprecipitated with the rabbit anti-GFP antibody from extracts of GFP-β2-syntrophin INS-1 cells labeled with ^32^P kept in culture media. A control immunoprecipitation from the same cells was performed using rabbit control IgG. The right panel shows the immunoblot with the anti-GFP antibody on the same immunoprecipitated material visualized by autoradiography. **C**) Domain structure of β2-syntrophin, including the phosphoserines identified by mass spectrometry. PH =  Pleckstrin Homology domain, PDZ  =  PSD95/Dlg/ZO-1 domain, SU  =  Syntrophin Unique domain.

Previous data suggested that β2-syntrophin is a phosphoprotein and that changes in its phosphorylation affect the interaction with ICA512, and thus granule traffic (Fig. 1B) [16]. The levels of the top migrating species of immunoprecipitated GFP-β2-syntrophin, similar to the corresponding species of β2-syntrophin, were reduced following the treatment of INS-1 cell extracts with alkaline phosphatase (Fig. 4A). Opposite changes were observed in INS-1 cells incubated prior to extraction with the phosphatase inhibitor okadaic acid (Fig. 4A). Immunoprecipitation from extracts of 32P-labeled GFP-β2-syntrophin INS-1 cells with the anti-GFP antibody, but not with control IgG, allowed the autoradiographic detection of multiple 32P-GFP-β2-syntrophin species, thus formally demonstrating their phosphorylation (Fig. 4B).

Analysis by nanoflow reversed phase chromatography and tandem mass spectrometry (nano LC-MS/MS) indicated that mouse GFP-β2-syntrophin immunoprecipitated from okadaic acid-treated INS-1 cells was phosphorylated on serine-75 (S75), -90 (S90), -213 (S213) and -373 (S373) (Fig. 4C), as independently observed in another cell type [37]. These serines are all flanked by proline-rich motifs that are conserved in the human and the predicted rat paralogue (Fig. S5). In mouse β2-syntrophin S75, S90 and S213 are adjacent to the PDZ domain and thus could affect the binding to ICA512, while S373 is located in the SU domain, and thus might regulate the interaction with utrophin.

**Figure 5:**
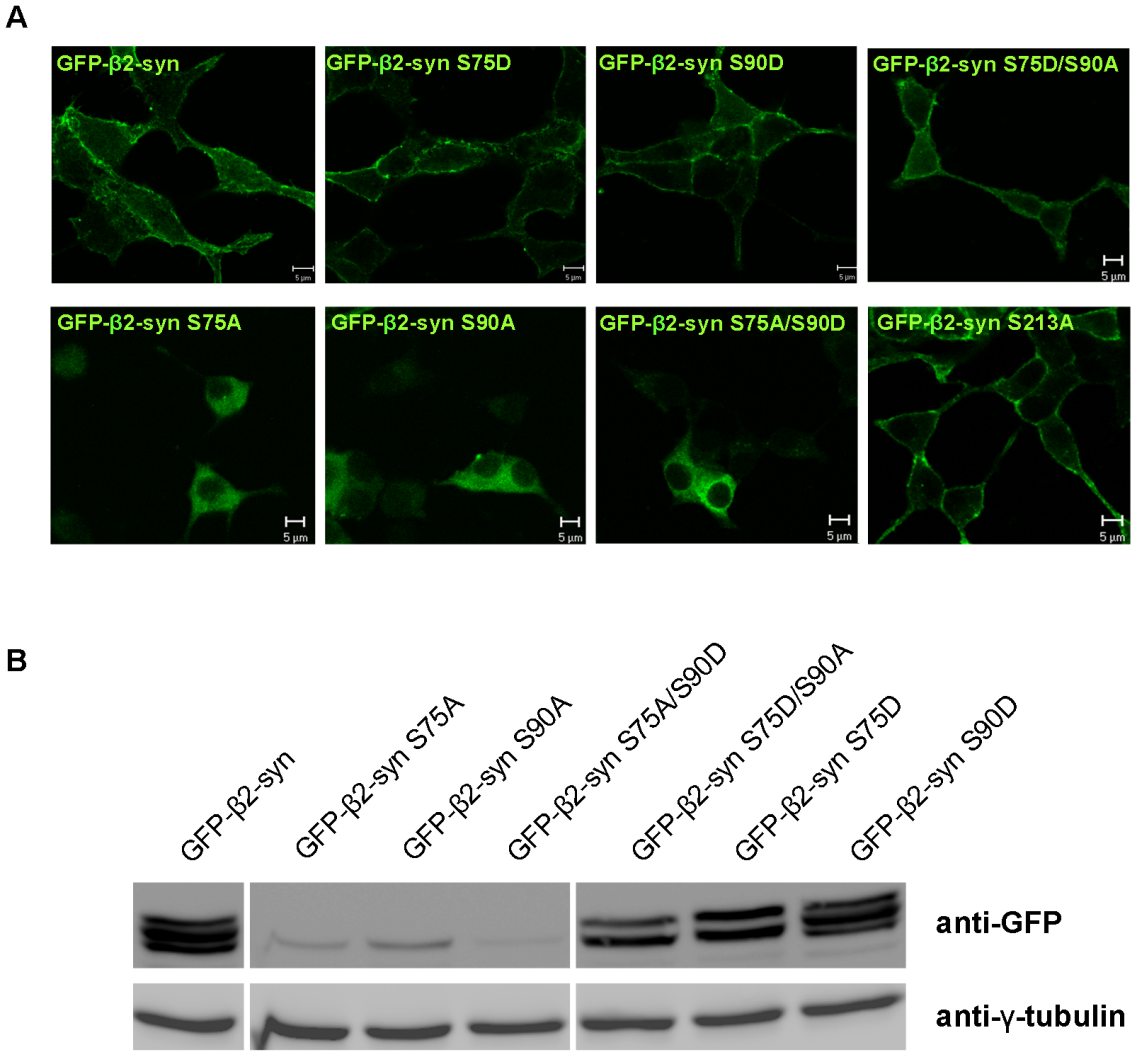
Characterization of β2-syntrophin phosphomutants. **A**) Confocal microscopy images of GFP-β2-syntrophin and mutants with single or dual replacements of serine (S) 75, 90 and 213 with either aspartate (D) or alanine (A). Bars: 5 µm. **B**) Expression pattern of GFP-β2-syntrophin variants in INS-1 cells as detected by western blotting with the anti-GFP antibody.

To gain insight into the role of their phosphorylation, each of these serines was independently replaced either with an aspartate (S/D), to mimic phosphorylation, or an alanine (S/A), to prevent it. The corresponding GFP-mutants were expressed in INS-1 cells (Fig. 5A–B and Fig. S6A–B). Similarly to GFP-β2-syntrophin, all single S/D mutants as well as the S213A mutant and the S75D/S90D and S75D/S90A double mutants were restricted to the cell cortex (Fig. 5A and Fig. S6A). The remaining single S/A mutants as well as the S75A/S90A and S75A/S90D double mutants were instead diffuse throughout the cytosol. Intriguingly, in cells expressing the S75A, S90A, and the S75A/S90D mutants the cortical fluorescence for F-actin and insulin seemed to be reduced compared to INS-1 and GFP-β2-syntrophin INS-1 cells (Fig. S7 and not shown).

By immunoblotting the pattern of all GFP-β2-syntrophin S/D mutants was similar to that of GFP-β2-syntrophin, except for the S75D mutants, which lacked one species (Fig. 5B and Fig. S6B). Strikingly, all GFP-β2-syntrophin S/A mutants, except S213A and S75D/S90A, appeared as weak single bands with mobility similar to that of the fastest migrating species of GFP-β2-syntrophin. The localization and/or electrophoretic changes caused by mutations of S75, S90 and S373 point to phosphorylation of these sites being significant, while modulation of S213 seems less relevant. Especially critical appears to be the phosphorylation/dephosphorylation cycling of S75, as replacement of this serine with either D or A reduced the SDS-PAGE pattern of the protein to either two or one species, respectively. Evidence that the S75D/S90A mutant, but not the S75A/S90D, localizes at the cell cortex and migrates as two species instead of one further suggests that regulation of S75 phosphorylation is dominant relative to that of S90 (Fig. 5B).

### β2-syntrophin S75 and S90 phosphorylation modulates the binding to ICA512, granule mobility and insulin secretion

**Figure 6:**
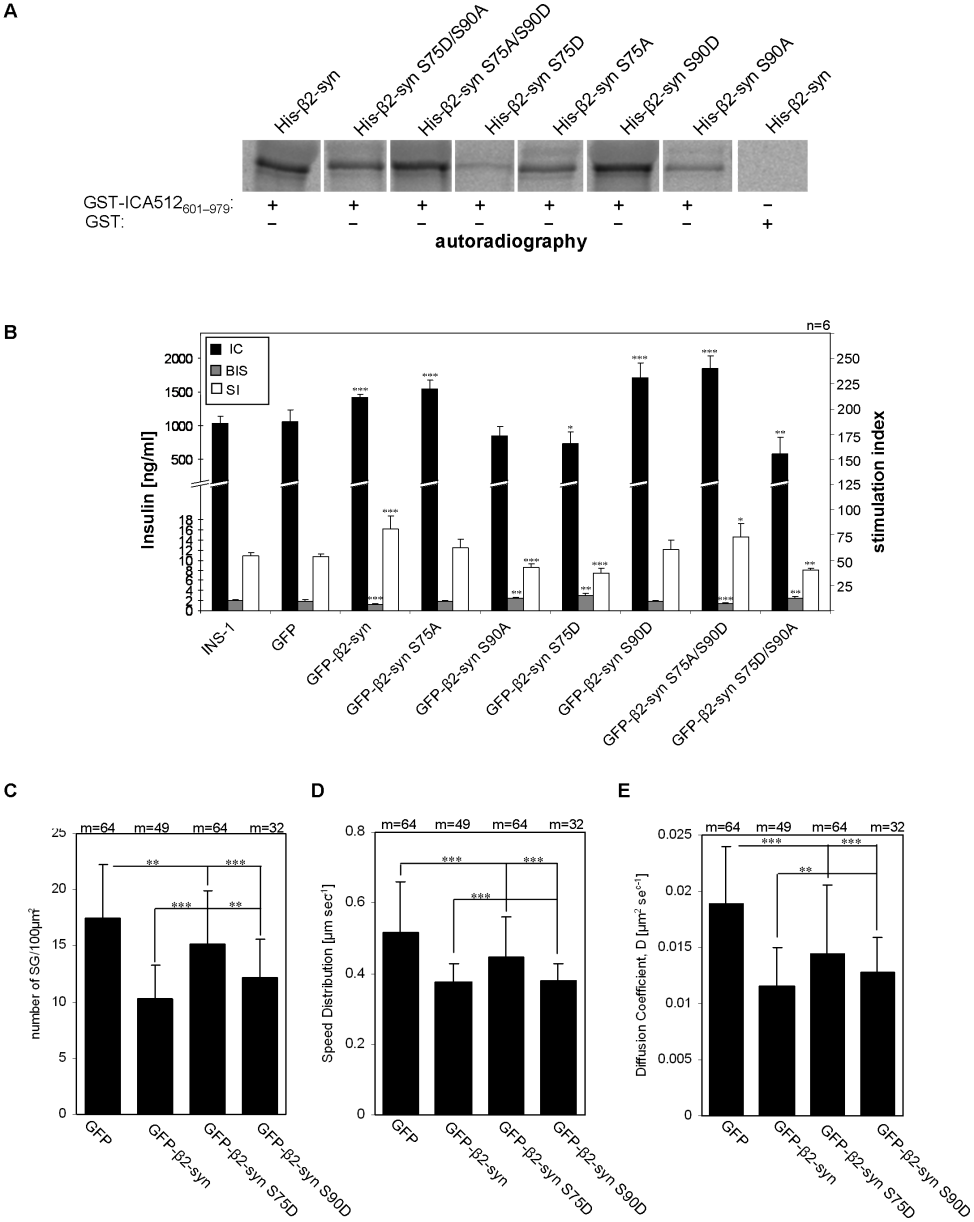
Binding of β2-syntrophin phosphomutants to ICA512 and impact on granule density, mobility and insulin secretion. **A**) Autoradiography of *in vitro* transcribed-translated ^35^S-labeled His-β2-syntrophin (His-β2-syn) S/D and S/A mutants following pull down assay with GST-ICA512_601–979_ or GST. **B**) Insulin content (IC), basal insulin secretion (BIS) and insulin secretion Stimulation Index (SI) of INS-1 cells transfected with the S75 and S90 GFP-β2-syntrophin mutants. Wild-type and GFP INS-1 cells were used as controls. n =  number of independent experiments; *, p = 0.05; **, p = 0.01; ***, p = 0.005; p-values of IC, BIS, and SI were calculated relative to those in INS-1 cells. **C–E**) Density (C), mean speed velocity (D) and diffusion coefficient (E) of CgB-mRFP1^+^ granules in resting INS-1 cells expressing GFP, GFP-β2-syntrophin or the GFP-β2-syntrophin S75D and S90D phosphomutants. m =  number of movies examined for each condition from six independent experiments.

We analyzed whether in vitro transcribed-translated His-tagged wild-type, S/D and S/A β2-syntrophins labeled with 35S-methionine differed in their ability to bind to the cytoplasmic domain of ICA512 (ICA512601–979) expressed as a GST-fusion protein in bacteria (Fig. 6A). Quantifications of autoradiograms indicated that GST-ICA512601–979 pulled down S75A, S90D and S75A/S90D His-β2-syntrophin more efficiently than S75D, S90A and S75D/S90A His-β2-syntrophin (Fig. S6C and Table S4). Mimicking the phosphorylation of S75 and S90 had opposite effects on the binding to GST-ICA512601–979, while mutations of S213 and S373 had no effect on it.

Next, variants of mouse β2-syntrophin with substitutions at serines 75 and 90 were transiently expressed in INS-1 cells to investigate their impact on insulin content and release (Fig. 6B and Table S1C). Similarly to the overexpression of GFP-β2-syntrophin, the overexpression of variants with increased binding to ICA512, such as S75A, S90D and S75A/S90D, correlated with increased insulin content relative to control INS-1 cells. Overexpression of the S75A/S90D mutant correlated also with reduced basal insulin secretion and increased HGHK stimulated insulin secretion. Conversely, overexpression of variants with reduced binding to ICA512, including S75D and S75D/S90A, correlated with the reduction of both insulin content and stimulated secretion, while basal insulin secretion was increased. In cells overexpressing the S90A mutant insulin content was not reduced, but basal secretion was increased and stimulated secretion was reduced. Finally, no significant changes in insulin content and secretion were observed in cells overexpressing GFP-β2-syntrophin constructs carrying mutations with opposite effects on the binding to ICA512, such as the S75A/S90A and S75D/S90D mutants (Table S1C).

Analysis by TIRFM further indicated that in resting CgB-mRFP1 INS-1 cells expressing GFP-β2-syntrophin S75D density and mobility of cortical granules were significantly increased by 47.6% (p =  1.65E-10) and 18% (p = 1.7E-04), respectively, relative to those expressing GFP-β2-syntrophin (Fig. 6C, D and Table S2A, B). The diffusion coefficient of granules in resting CgB-mRFP1 INS-1 cells expressing GFP-β2-syntrophin S75D was also significantly increased by 26% (p = 0.0019) compared to those expressing GFP-β2-syntrophin (Fig. 6E and Table S2C). Conversely, the density and mobility of cortical granules in CgB-mRFP1 INS-1 cells overexpressing either GFP-β2-syntrophin or GFP-β2-syntrophin S90D were similarly reduced relative to cells expressing GFP only. Evidence that overexpression of a β2-syntrophin phosphomutant with reduced binding to ICA512 correlated with increased mobility of cortical granules in resting cells is consistent with the hypothesis that phosphorylation of β2-syntrophin affects the ability of the cortical cytoskeleton to restrain granules.

### Cdk5 phosphorylates β2-syntrophin at S75

**Figure 7:**
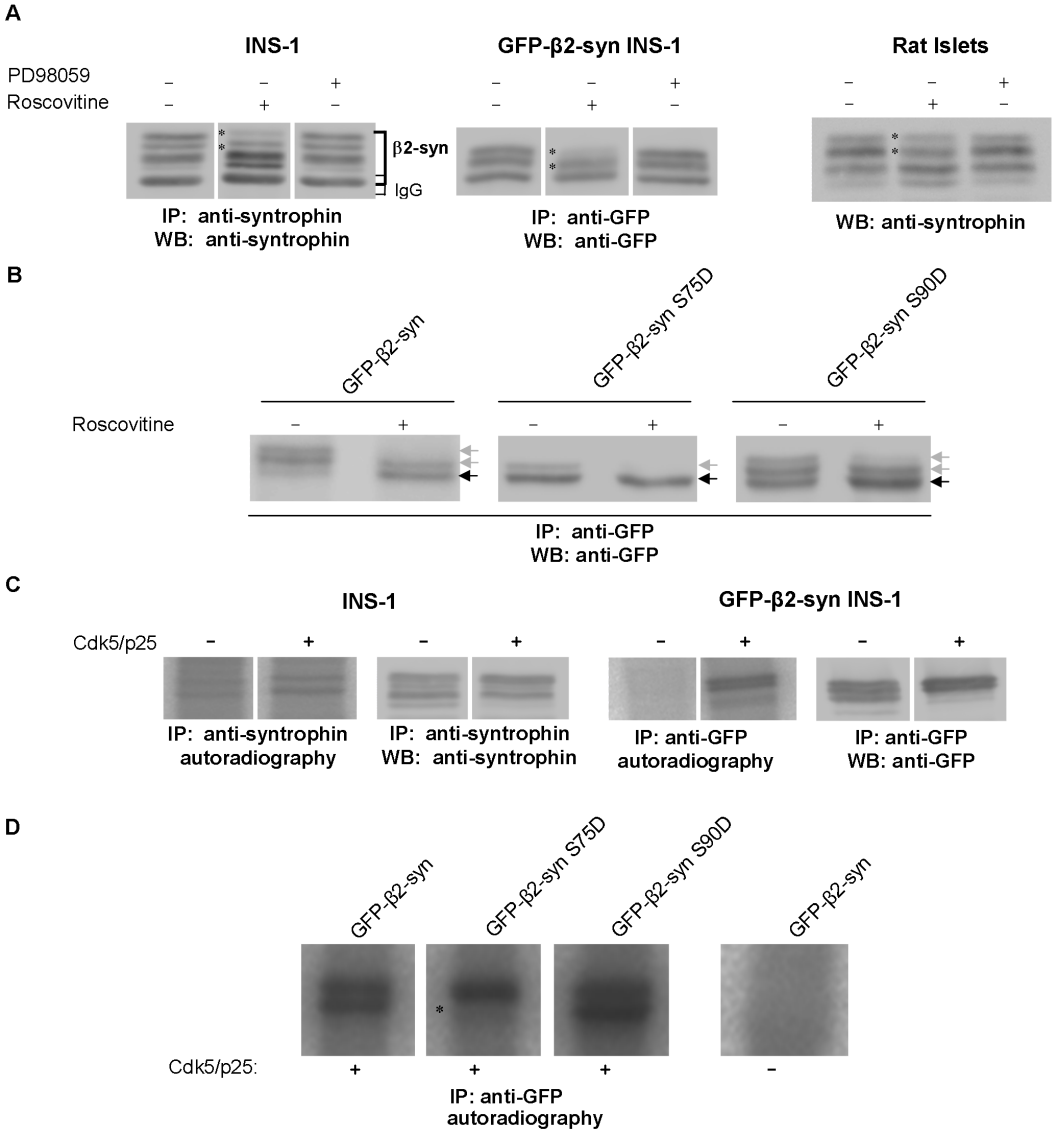
Phosphorylation of endogenous and GFP-β2-syntrophin and the S/D mutants by Cdk5. **A**) Immunoblots with anti-syntrophin or anti-GFP antibodies on rat islet extracts (right panel) or immunoprecipitates obtained with the same antibodies from extracts of INS-1 cells (left panel) or GFP-β2-syntrophin INS-1 cells (middle panel) treated with the Cdk5 inhibitor roscovitine or the Erk1/2 inhibitor PD98059. Asterisks indicate the β2-syntrophin species sensitive to roscovitine. **B**) Immunoblots with the anti-GFP antibody on immunoprecipitates obtained with the same antibody from extracts of INS-1 cells expressing GFP-β2-syntrophin variants and treated with roscovitine. Gray and black arrows point to β2-syntrophin species the levels of which decreased or increased in response to roscovitine, respectively. **C**) Autoradiographies and immunoblots of β2-syntrophin (left panels) and GFP-β2-syntrophin (right panels) immunoprecipitated with anti-syntrophin or anti-GFP antibodies from INS-1 and GFP-β2-syntrophin INS-1 cells, respectively. Immunoprecipitates were incubated with or without the Cdk5/p25 complex in the presence of ^32^P-γ-ATP. **D**) Autoradiography of GFP-β2-syntrophin variants immunoprecipitated with the anti-GFP antibody from GFP-β2-syntrophin INS-1 cells and incubated with the Cdk5/p25 complex as in C. An asterisk indicates the β2-syntrophin species that is lacking in the GFP-β2-syntrophin S75D mutant.

S75 and S90 conform to the consensus for phosphorylation by the proline-directed kinases Cdk5, which modulates insulin secretion [38] and Erk 1/2. Thus, we tested whether the Cdk5 inhibitor roscovitine and the Erk1/2 inhibitor PD98059 affected β2-syntrophin phosphorylation. Roscovitine, but not PD98059, altered the electrophoretic pattern of endogenous β2-syntrophin of INS-1 cells and rat pancreatic islets as well as all tested GFP-β2-syntrophin variants overexpressed in INS-1 cells (Fig. 7A, B, Fig. S8A and not shown). Upon roscovitine treatment, in particular, the slower migrating species of the S75D mutant became undetectable. Moreover, the purified Cdk5/p25 complex phosphorylated in vitro two slow migrating species of endogenous and GFP-β2-syntrophins immunoprecipitated from INS-1 cells (Fig. 7C). The levels of these two β2-syntrophin species, and especially the slowest migrating form, were increased upon incubation with the Cdk5/p25 complex. Notably, one of these species phosphorylated by Cdk5/p25 was selectively missing in the case of the S75D mutant, but not in the case of the other tested GFP-β2-syntrophin variants (Fig. 7D and not shown). Taken together, these results demonstrate that Cdk5 phosphorylates β2-syntrophin on S75 and on another site, which remains to be determined.

**Figure 8:**
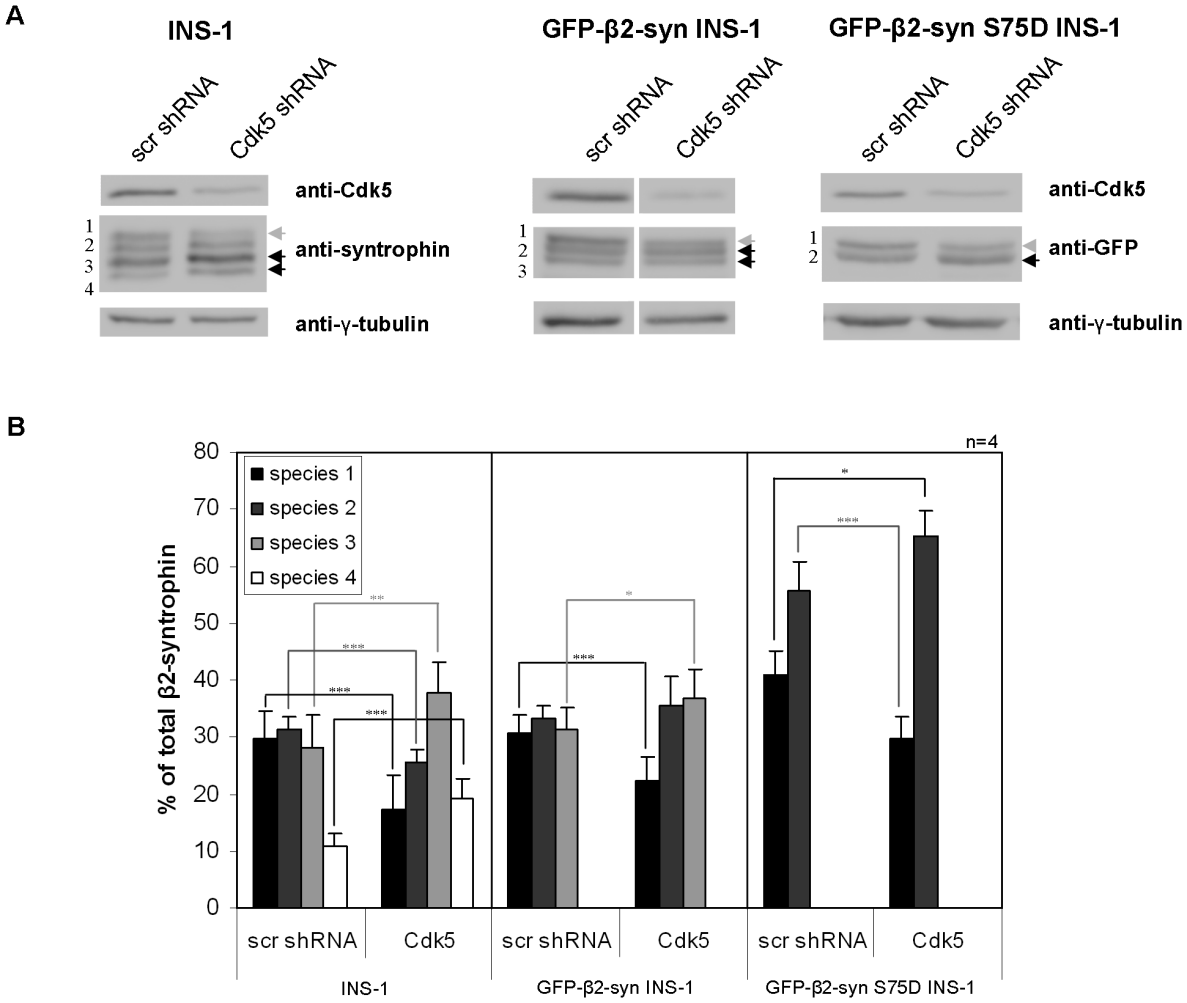
Expression of β2-syntrophin species upon Cdk5 depletion. **A**) Immunoblots with anti-syntrophin, anti-Cdk5 and anti-γ-tubulin antibodies on extracts of INS-1 (left panels), GFP-β2-syntrophin INS-1 (middle panels) and GFP-β2-syntrophin S75D INS-1 (right panels) cells following the knockdown of Cdk5 (Cdk5 shRNA). As control, INS-1 cells were transfected with a scrambled shRNA. Gray and black arrows point to the β2-syntrophin species the levels of which decreased or increased upon Cdk5-depletion, respectively. The β2-syntrophin species were numbered from top to bottom **B**) Quantification of the immunoblots shown in A. p-values are relative to cells transfected with scr shRNA. n =  number of experiments. *, p = 0.05; **, p = 0.01; ***, p = 0.005.

To further verify this conclusion, the expression of Cdk5 was knocked down in control INS-1 cells and in INS-1 cells expressing GFP-β2-syntrophin or the S75D mutant. Upon reduction of Cdk5 mRNA and protein by >70% the levels of the two slower migrating species of β2-syntrophin were decreased in comparison to control cells, while those of the faster migrating species were increased (Fig. 8A and B). The decrease of the top migrating species was also significant in the case of GFP-β2-syntrophin S75D; supporting the possibility that β2-syntrophin contains additional Cdk5 phosphorylation sites. Furthermore, the pattern changes induced by the down regulation of Cdk5 were more pronounced in cells stimulated with HGHK than in resting cells (Fig. S8B). These results corroborate the conclusion that stimulation of insulin secretion triggers the Cdk5-dependent phosphorylation of β2-syntrophin.

## Discussion

Knowledge about the interaction of the cortical cytoskeleton with insulin granules, and secretory granules in general, is rapidly accumulating. The best-defined complex for this interaction includes the granule-associated GTPase Rab27 and its effectors Slac-2c/MYRIP [39], [40] and melanophilin [41], [42]. Slac-2c/MYRIP and melanophilin bind to F-actin [42], [43] and the motor protein myosin Va [43], [44], which drives the transport of secretory granules along cortical actin microfilaments [6], [45]. Myosin Va has also be implicated in their anchoring to the plasma membrane [46].

Interaction of the cytoplasmic domain of the granule transmembrane protein ICA512 with the multiarm adaptor β2-syntrophin has been proposed as an additional mechanism for the tethering of insulin granules to the actin cytocortex [14]. Previous data indicated that INS-1 cells express several β2-syntrophin species, which conceivably originate from phosphorylation/dephosphorylation at various sites [16]. Stimulation of INS-1 cells with HGHK changes the ratio among β2-syntrophin species and modulates the binding of its PDZ domain to ICA512 [16], [47].

Here we demonstrate that multiple β2-syntrophin species are also present in pancreatic islets and that the expression levels and phosphorylation state of β2-syntrophin modulate insulin secretion in INS-1 cells. Specifically, we show that the expression of β2-syntrophin positively correlates with insulin content and stimulated secretion, while it inversely correlates with basal insulin secretion. Hence, the overexpression of β2-syntrophin attenuates the typically “leaky” phenotype of INS-1 cells relative to primary β-cells [48]. Overexpression of β2-syntrophin was shown to increase the levels of ICA512 [47]. This observation is in agreement with the increased number of granules in β2-syntrophin overexpressing cells.

Overexpression of β2-syntrophin diminishes the diffusion and short-range movements of granules in resting cells. Greater insulin stores and reduced granule mobility in resting cellsoverexpressing β2-syntrophin are consistent with the hypothesis that this protein tethers granules to the cortical cytoskeleton. Changes in the phosphorylation of β2-syntrophin in response to glucose stimulation may weaken the anchoring of granules to microfilaments, thereby enabling a larger number of vesicles to be mobilized and released. This interpretation is corroborated by the increased frequency of pear-shaped granules in sub-maximally stimulated, but not in resting, INS-1 cells overexpressing β2-syntrophin relative to control cells. This shape may result from the force applied by a motor to the granule pole proximal to the transport cable [49]-[51]. Alternatively, it could reflect the compression generated by the focal polymerization of actin at the rear end of moving granules, as it has been previously described for the transport of endosomes and lysosomes [50], [52]–[55]. In view of these considerations, it will be especially interesting to assess the relationship of β2-syntrophin with the machinery for granule transport. Silencing of myosin Va, similarly to the silencing of Slac-2c/MYRIP [40], impairs the glucose stimulated recruitment and exocytosis of insulin granules, although it had no impact on basal insulin secretion [5]. Slac-2c/MYRIP overexpression led also to impaired granule mobility and hormone secretion in PC12 cells, whereas a construct lacking the actin-binding domain increased mobility while still impairing secretion [39]. These data raised the hypothesis that myosin Va-mediated translocation of granules only occurs after detachment of Slac-2c/MYRIP from microfilaments, possibly due to the calpain-mediated cleavage of Slac-2c/MYRIP actin-binding C-terminus [56]. Intriguingly, calpain disrupts also the binding of ICA512 to β2-syntrophin, and thereby to actin [47]. Thus, at least two distinct complexes regulate the tethering and release of granules from the cortical cytomatrix. Importantly, regulation of the ICA512/β2-syntrophin complex by phosphorylation rather than calpain cleavage provides a mechanism for reversible granule tethering to F-actin.

The density of cortical granules detected by TIRFM in resting GFP-β2-syntrophin INS-1 cells was reduced relative to resting cells expressing GFP alone. This observation is only apparently in contrast with the biochemical and microscopy data indicating that β2-syntrophin overexpression increases insulin stores and the total number of granules. Recent independent observations by the Nagamatsu and the Seino laboratories using a modified set-up for TIRFM suggests that immobile insulin granules accumulate in a zone situated 400–500 nm from the plasma membrane (personal communication), i.e. at a distance that is beyond the optical depth of conventional TIRFM (≤200 nm). By trapping more granules in this zone, β2-syntrophin could prevent their access to the region visualized here by TIRFM. This interpretation, however, does not explain the reduced mobility of cortical granules in β2-syntrophin overexpressing cells. To clarify this point, further studies will be necessary regarding the relationship of β2-syntrophin with the granule transport machinery.

Our data confirm that β2-syntrophin is a phosphoprotein and demonstrate that its complex phosphorylation pattern changes in relation to stimulation of insulin secretion. The conserved S75 and S90 adjacent to the PDZ domain are especially relevant as these phosphosites modulate the intracellular compartmentalization of β2-syntrophin and its interaction with ICA512. Specifically, phosphorylation of S75 and dephosphorylation of S90 weaken the binding to ICA512 in vitro. This could account for the reduced insulin content and increased basal insulin secretion of INS-1 cells expressing the β2-syntrophin mutants S75D, S90A and S75D/S90A. Furthermore, overexpression of the β2-syntrophin S75D phosphomutant, but not β2-syntrophin S90D enhanced the mobility of cortical granules relative to resting cells overexpressing β2-syntrophin. These data corroborate the hypothesis that β2-syntrophin restrains granule mobility at rest and that its phosphorylation/dephosphosphorylation represents a regulatory mechanism modulating actin/granule interaction and therefore insulin secretion. Evidence that all S75A and S90A mutants, except S75D/S90A, were diffusely distributed in the cytosol rather than being restricted to the cell cortex further suggest that cyclic phosphorylation/dephosphorylation of these serines, and especially of S75, is critical for the proper localization and function of β2-syntrophin.

We show that Cdk5 phosphorylates β2-syntrophin on S75. In neurons Cdk5 can either increase or decrease neurotransmitter release by phosphorylating various proteins implicated in exocytosis of synaptic vesicles, including synapsin I [57], Munc18 [58], and P/Q type voltage-dependent Ca2+-channels [59]. Cdk5 appears to play contrasting roles in regards to synaptic vesicle exocytosis [58], [59], endocytosis [60], [61] and insulin secretion [62], [63]. The stimulatory role of Cdk5 and its activating cofactor p39 on insulin secretion has been attributed to the phosphorylation of Munc18 [62], whereas its inhibitory effect has been ascribed to the phosphorylation of a serine in the αc1 subunit of the L-type voltage-dependent Ca2+-channels [63], which regulates the binding of the SNARE proteins syntaxin and SNAP25 to these channels. As for synaptic vesicle endocytosis [64], these apparently conflicting roles of Cdk5 in β-cell secretion can be explained in view of our present findings. Cdk5-mediated phosphorylation of β2-syntrophin on S75 could indeed enhance insulin secretion by promoting the mobilization of cortical granules. Dephosphorylation of S75, on the other hand, would enable the cortical cytomatrix to recapture granules that did not undergo exocytosis, thereby retaining them at a favorable distance for recruitment in the next round of exocytosis.

In conclusion, these data extend and partially revise our previous model wherein β2-syntrophin affects the traffic and exocytosis of insulin granules by mediating their anchoring to the cortical cytoskeleton through its binding to ICA512 [16]. This interaction is dynamically controlled via phosphorylation/dephosphorylation of β2-syntrophin at multiple serines. Among them, S75, which is phosphorylated by Cdk5, appears to play a dominant role both in the cortical localization of β2-syntrophin and the association with ICA512. Understanding the dynamics of this interaction will require the structural resolution of the β2-syntrophin/ICA512 complex as well as the identification of other kinases, phosphatases and factors that regulate this association.

## Materials and Methods

### Islet isolation and Cell culture

Pancreatic islets from female Wistar rats were isolated and cultured [65] and INS-1 cells were grown [66] as described. Briefly the islets standard culture medium contained 5.5 mM (islets) glucose, while the medium for INS-1 cells contained 11 mM glucose. Unless otherwise stated, INS-1 cells and pancreatic islets were directly harvested from culture media or incubated for 60 min in resting buffer (0 mM glucose, 5 mM KCl) and then for 120 min in fresh resting or stimulating (25 mM glucose (HG) and/or 55 mM KCl (HK)) buffer, as described [16]. Cells were treated for 60 min with 40 µM roscovitine (Alexis Biochemicals), 5 µM PD98059 (Calbiochem) or 30 min with 1 µM okadaic acid (Alexis Biochemicals) prior to harvest.

### cDNA constructs

The following constructs have been already described: mouse GFP-β2-syntrophin [18], His-β2-syntrophin [15], GST-ICA512601–979 [15]. Single and double mutants of β2-syntrophin in which serines S75, 90, 213 and 373 were replaced either individually or in tandem by aspartate (S/D) or alanine (S/A) were generated using mouse β2-syntrophins N-terminally fused to GFP or His-tag as templates for mutagenesis with the QuikChange Multi Site-Directed Mutagenesis Kit (Stratagene). The oligonucleotides used for mutagenesis are listed in Table S5A. The human cDNA of the granule cargo chromogranin B (CgB; gift from W. Huttner, MPI-CBG, Dresden) was fused in frame at the 5′-end of monomeric Red Fluorescent Protein (mRFP1) cDNA (Planetgene; [67]). All constructs were generated using conventional procedures and verified by DNA sequencing.

### Cell transfections

Electroporation of INS-1 cells and generation of stable GFP- and GFP-β2-syntrophin INS-1 cell clones were performed as described [68]. CgB-mRFP1 INS-1 clones were selected for puromycin resistance, and further screened by fluorescence microscopy and immunoblotting for expression of the transgene.

### Cell extracts, Western blottings

Pancreatic islets and INS-1 cells were washed with ice cold PBS, and extracted in lysis buffer (PBS pH7.5, 1% Triton X-100, 1 mM EDTA, 1% protease and phosphatase inhibitor) at 4°C [16]. Protein measurements and immunoblottings were performed as described [68] using the following primary antibodies: monoclonal mouse anti-syntrophin 1351 [69], anti-γ-tubulin (Sigma) and anti-Cdk5 (Molecular Probes) antibodies; polyclonal goat (gift from David Drechsel, MPI-CBG, Dresden) and rabbit (Molecular Probes) anti-GFP antibodies. Chemiluminescence was developed and quantified as described [68].

### Immunoprecipitation

Protein extracts of INS-1 cells were incubated overnight at 4°C with one of the following antibodies: anti-syntrophin, goat or rabbit anti-GFP, goat or rabbit IgGs (Sigma), followed by incubation with protein G or A sepharose (GE Healthcare Biosciences) for 120 min. The beads were washed 10 times, and loaded on a 10% SDS-PAGE followed by western blotting. For dephosphorylation G sepharose-coupled immunoprecipitates of endogenous or GFP-β2-syntrophins were equilibrated with dephosphorylation buffer containing 50 U alkaline phosphatase (Roche) for 1 hour at 37°C.

### *In vitro* phosphorylation

GFP-β2-syntrophin INS-1 cells were pre-incubated for 30 min. in phosphate-free medium. Cells were equilibrated in phosphate-free medium containing 25 mCi 32P per dish (0.7 mCi/ml) for 3 hours and immunoprecipitated as described above. Following western blotting, 32P-labeling of immunoprecipitated GFP-β2-syntrophin was detected by autoradiography.

### *In vitro* kinase assay

The Cdk5/p25 complex was expressed and purified from Sf9 cells co-infected with baculovirus as described [70]. Phosphorylation of immunoprecipitated β2-syntrophin from INS-1 cells or GFP-β2-syntrophin from GFP-β2-syntrophin INS-1 cells or the transiently expressed phosphomutants in the presence of 10 µCi γ-[32P]ATP was performed as described [71]. Kinase reactions were stopped by adding SDS-PAGE sample buffer. Samples were analyzed by western blotting and autoradiography.

### Pull-Down Assays

Full-length His-β2-syntrophin constructs were *in vitro* transcribed-translated with the T7-coupled transcription/translation system (Promega). GST and GST-ICA512601–979 were expressed in bacteria as described [16]. 35S-methionine-labeled His-β2-syntrophin variants were then equilibrated with 5 µg of GST or GST-ICA601-979 coupled to glutathione-sepharose beads (GE) and incubated 4 hr at RT. Beads were washed 10 times with binding buffer and eluted with 10 mM of reduced glutathione. Eluted proteins were subjected to SDS-PAGE and analyzed by autoradiography.

### RNA interference

The pGENE-CLIP U1 cassette system (Promega) was used according to the manufacturers instruction to clone short hairpin (sh)RNAs for Cdk5, GFP, rat β2-syntrophin (β2-syn) or scrambled (scr) (Table S5B). INS-1 cells were transfected with shRNA by electroporation as described [68]. Cells were harvested 4 days after transfection and knockdown of Cdk5 and β2-syntrophin was measured by qRT-PCR and western blotting [68].

### Insulin RIA

Insulin content and secretion were assessed with the Sensitive Rat Insulin RIA Kit (Linco Research) as described [68]. The insulin stimulation index was calculated as follows: secreted/total insulin in stimulated conditions vs. secreted/total insulin in resting conditions.

### Immunocytochemistry

Labeling of transfected or non-transfected INS-1 cells was performed as described [68]. The following primary and secondary antibodies were used: anti-syntrophin, mouse anti-insulin (Sigma), Alexa488- and Alexa568-conjugated goat-anti-mouse or -rabbit IgGs (Molecular Probes). Phalloidin-rhodamine (Molecular Probes) was used to visualize actin filaments and nuclei were counterstained with DAPI (Sigma). Images of 0.5 µm optical sections were acquired with an inverted confocal microscope LSM Meta 405 nm (Zeiss) using a 63x oil immersion objective with a numerical aperture of 1.4.

### TIRFM

Prior to TIFM imaging (see Methods S1), INS-1 cells stably expressing mouse CgB-mRFP1 were grown in an open chamber, incubated in resting or stimulated media and transferred onto a thermostat-controlled stage at 37°C. CgB-mRFP1+ granules in INS-1 cells expressing GFP-β2-syntrophin or GFP were visualized by excitation of mRFP1 and GFP. Images were collected using the IQ 1.7 software (Andor Technology) with an exposure time of 40 ms (sequential imaging of GFP and mRFP1, each 20 ms), for a total of 1200 frames (0.14 sec/frame), each movie in resting or stimulated conditions. Automated image analysis was performed with the MotionTracking/Kalaimoscope software (Transinsight GmbH), as described (Methods S1; [72]). Statistical data for granule density in GFP/CgB-mRFP1 and GFP-β2-syntrophin/CgB-mRFP1 cells were obtained from 15 and 17 movies at rest and 15 and 12 movies in stimulated conditions, respectively (Fig. 3). For speed measurements the total number of experiments and tracked CgB-mRFP1+ granules in GFP and GFP-β2-syntrophin/CgB-mRFP1 cells at resting was 15/18564 and 17/19176 and in stimulated conditions 5/4367 and 8/4131 (Fig. 3). Statistical data for granule density or mobility in CgB-mRFP1 INS-1 cells overexpressing GFP, GFP-β2-syntrophin GFP-β2-syntrophin S75D or GFP-β2-syntrophin S90D were obtained from 64, 49, 64 or 32 movies at rest, respectively (Fig. 6).

### Electron microscopy (EM)

INS-1 cells were subjected to 3 different protocols for EM. In protocol 1 cells were grown on glass coverslips for four days, fixed with 1% glutaraldehyde in 0.1 M cacodylate buffer and processed for standard Epon-embedding [73]. In protocol 2 cells were grown on sapphire discs and high pressure frozen and freeze substituted as described [74]. For cryoimmuno EM (protocol 3) cells were fixed and scraped into a pellet as described [75]. Sections were incubated with anti-insulin (Sigma) antibody followed by goat anti-mouse IgG conjugated to 12 nm gold particles (Dianova). Images of cells kept in culture media or rested for 180 min prior to fixation with aldehydes (protocol 1) were used for the morphometry analysis of granules. Statistics was performed with the ImageJ Software (http://rsb.info.nih.gov/ij/index.html). An ellipse was fitted on the granules, the major and minor diameters were measured and the number of granules per cell was counted.

### Mass Spectrometry, Phosphomapping

SDS-PAGE separated proteins were subjected to in-gel digestion as described previously [76]. Automated nanoflow LC Electrospray-Ionisation (ESI) MS/MS analysis was performed using a QTOF Ultima mass spectrometer (Waters) employing automated data dependent acquisition [77]. Raw data was processed using ProteinLynx Global Server ProteinLynx (V2.0) into pkl peak list files and searched against the NCBI non-redundant protein database using an in-house MASCOT server version 2.0 (Matrix Sciences). The following constraints were applied: potentially upto two missed cleavage sites were allowed; ±0.5 Da tolerance for MS and ±0.2 Da for MS/MS fragment ions; carbamidomethyl cysteine was specified as a fixed modification, deamidation (NQ) phosphorylation (S, T, Y) and methionine oxidation (M) were specified as variable modifications.

### Sequence Alignment, Motifscan

Multiple sequence alignments were produced with the program ClustalW (http://www.ebi.ac.uk/clustalw/) and manually re-fined. Protein domains of β2-syntrophin were located with Scanprosite (http://au.expasy.org/tools/scanprosite/) and adapted from the literature [15], [19]. Prediction of kinase binding sites was performed with http://scansite.mit.edu/motifscan_seq.phtml.

### Statistics and Graphics

Quantification of western blots and autoradiographies was performed using Image Gauge V3.45 (Fuji, Tokyo, Japan). Statistics was performed using the unpaired Student's t-test. Results are presented as mean ± SE from at least three independent experiments. Histograms were prepared with Microsoft Excel (Microsoft).

## Supporting Information

Methods S1 (0.04 MB DOC)

Figure S1 Expression pattern of β2-syntrophin in rat INS-1 cells and in rat and mouse islets. A) Immunoblotting (left panels) with anti-syntrophin and anti-γ-tubulin antibodies on extracts of INS-1 cells, rat and mouse islets kept in their standard culture medium with either 11 mM (INS-1 cells) or 5.5 mM (islets) glucose. Quantification (right histogram) of the immunoblots shown on the left. B) Immunoblotting with anti-syntrophin antibody on INS-1 cells (left panel) and rat islets (right panel). Cells were previously kept at rest (0 mM glucose, 5 mM KCl) or stimulated with high (25 mM) glucose and low (5 mM) KCl (HGLK), low (0 mM) glucose and high (55 mM) KCl (LGHK) or high glucose and high KCl (HGHK).
(0.33 MB TIF)

Figure S2 Impact of β2-syntrophin knockdown on insulin content and secretion. Insulin content (IC), basal insulin release (BIS) and insulin secretion Stimulation Index (SI) of INS-1 cells following the depletion of β2-syntrophin by RNA interference (β2-syn shRNA) and its rescue with GFP-β2-syntrophin. Control cells were transfected in parallel with a scrambled shRNA, with or without GFP-β2-syntrophin. n =  number of independent experiments; *, p = 0.05; **, p = 0.01; ***, p = 0.005; p-values are relative to scr shRNA transfected INS-1 cells.
(0.10 MB TIF)

Figure S3 Colocalization of GFP-β2-syntrophin with F-actin and of CgB-mRFP1 with insulin. A) Confocal microscopy of GFP and GFP-β2-syntrophin INS-1 cells labeled with rhodaminated-phalloidin (pseudored). B) Co-localization of CgB-mRFP1 (pseudored) and insulin (pseudogreen) in stable CgB-mRFP1 INS-1 cells. Bars: 5 µm.
(2.29 MB TIF)

Figure S4 Granule morphometry in INS-1 cells and GFP-β2-syntrophin INS-1 cells. A) Electron microscopy images of INS-1 and GFP-β2-syntrophin INS-1 cells kept in culture medium and fixed with aldehydes (protocols 1 and 3) or by high-pressure freezing (HPF; protocol 2) followed by Epon-embedding (protocols 1 and 2) or freezing (Cryo; protocol 3) before sectioning. Aldehyde/cryo specimens were immunogold-labeled with an anti-insulin antibody. The black dots in the left panels correspond to immunogold-labeled insulin. White arrowheads point to granules with a deformed shape. B) Percentage of granules with a major/minor diameter ≥1.9 in resting or sub-maximally stimulated INS 1 and GFP-β2-syntrophin INS-1 cells. Data were obtained from 2 independent experiments (n). *, p = 0.05; C) Insulin secreted from INS-1 cells and GFP-β2-syntrophin INS-1 cells kept in culture medium, compared to resting and HGHK-stimulated cells. n =  number of independent experiments; *, p = 0.05; **, p = 0.01. p-values in are relative to INS-1 cells and, GFP-β2-syntrophin INS 1 cells kept in culture media.
(2.82 MB TIF)

Figure S5 Conservation of phosphoserines in rodent and human β2-syntrophin. Alignment of the primary amino acid sequences of mouse (mβ2-syn), human (hβ2-syn) and predicted rat (rβ2-syn) β2-syntrophin. The split PH1 domain (PH1a, PH1b) of β2-syntrophin is marked in italic and underlined; the PH2 domain is underlined; the PDZ domain is boxed, while the SU domain is in italic. The phosphoserines of mouse β2-syntrophin identified by mass spectrometry are marked in red, while the isolated phosphopeptides are in bold and underlined. The rat β2-syntrophin sequence was assembled from two in silico sequences (XM_344764, XP_226430) of the rat genome project. Asterisks mark amino acids that are identical in mouse and human; double dots mark conservative changes, while single dots mark semi-conservative changes.
(1.54 MB TIF)

Figure S6 Localization, expression pattern and ICA512-binding of GFP-β2-syntrophin mutants. A) Confocal microscopy images of INS-1 cells expressing the indicated single or double GFP-β2-syntrophin S/D and S/A mutants compared to GFP-β2-syntrophin and GFP INS-1 cells. Bars: 5 µm. B) Immunoblots with anti-GFP and γ-tubulin antibodies on extracts of INS-1 cells expressing the GFP-β2-syntrophin variants. The left panel was overexposed relative to the right panel in order to detect GFP-β2-syntrophin S75A/S90A and S373A. C) Ratio of *in vitro* transcribed-translated ^35^S-His-β2-syntrophin phosphomutants pulled down with GST-ICA512_601–979_ relative to ^35^S-His-β2-syntrophin. n =  number of independent experiments; *, p = 0.05; **, p = 0.01.
(0.74 MB TIF)

Figure S7 Colocalization of GFP-β2-syntrophin variants with the actin cytoskeleton and insulin. A) Colocalization of β2-syntrophin and GFP-β2-syntrophin variants with F-actin. B) Colocalization of β2-syntrophin and GFP-β2-syntrophin variants with insulin. Bars: 5 µm.
(2.59 MB TIF)

Figure S8 Impact of Cdk5 on the expression pattern of β2-syntrophin and GFP-β2-syntrophin variants in resting and stimulated INS-1 cells. A) Western blotting with anti-GFP, -syntrophin and -γ-tubulin antibodies on extracts from resting and HGHK-stimulated INS-1 cells (left panels) and INS-1 cells expressing different GFP-β2-syntrophin variants. Cells were treated or not with roscovitine prior to extraction. B) Immunoblots with anti-Cdk5, -GFP, -syntrophin, and -γ-tubulin antibodies on extracts of resting and HGHK-stimulated INS-1 (right panels), GFP-β2-syntrophin INS-1 (middle panels) and GFP-β2-syntrophin S75D INS-1 (left panels) cells following the knockdown of Cdk5 (Cdk5 shRNA). Control INS-1 cells were transfected with a scrambled shRNA.
(0.42 MB TIF)

Table S1 Insulin content and secretion of INS-1 cells relative to GFP-β2-syntrophin expression and phosphorylation. A) Insulin content (IC), basal (BIS) and Stimulation Index (SI) of GFP-β2-syntrophin INS-1 cell clones G1, G6 and G8 compared to INS-1 cells and GFP INS-1 cells. B) IC, BIS and SI of INS-1 cells transfected with scr shRNA or β2-syn shRNA, with or without GFP-β2-syntrophin. C) IC, BIS and SI of INS-1 cells and INS-1 cells transiently transfected with GFP or GFP-β2-syntrophin variants. The data in A, B and C are from 3, 4 and 6 independent experiments, respectively.
(1.07 MB PDF)

Table S2 Granule density and mobility in GFP-β2-syntrophin INS-1 cells. A) Density of granules/100 µm^2^. B) Granule mean speed distribution; C) Mean square displacement presented as diffusion coefficient. The data are from 4 and 6 independent experiments, respectively.
(0.97 MB PDF)

Table S3 Granule morphometry in INS-1 cells and GFP-β2-syntrophin INS-1 cells. Total number of measured granules, number and percent of granules with a major/minor granule diameter ≥1.9 from INS-1 and GFP-β2-syn INS-1 cells in resting (R) buffer or in culture media (M).
(0.21 MB PDF)

Table S4 Quantification of β2-syntrophin-ICA512 pull-down assays. Autoradiographic signals of *in vitro* transcribed-translated ^35^S-labeled His-β2-syntrophin S/D and S/A mutants recovered with GST-ICA512_601–979_ expressed as percentage of pull down His-β2-syntrophin.
(0.33 MB PDF)

Table S5 Primers for A) site-chain-mutagenesis, B) RNAi and C) qRT-PCR.
(0.69 MB PDF)

Movie S1 TIRFM movie tracking the motion of CgB-mRFP1^+^ granules in resting INS-1 cells transiently expressing GFP. The automated tracking of CgB-mRFP1^+^ granules is shown in the left panel of the movie. The signals for GFP and mRFP1 are separately shown in the middle and right panels, respectively. Bar  = 5 µm.
(6.11 MB MP4)

Movie S2 TIRFM movie tracking the motion of CgB-mRFP1^+^ granules in resting INS-1 cells transiently expressing GFP-β2-syntrophin. The automated tracking of CgB-mRFP1^+^ granules is shown in the left panel of the movie. The signals for GFP and mRFP1 are separately shown in the middle and right panels, respectively. Bar  = 5 µm.
(6.11 MB MP4)
