# Supplementary material for: β2-Syntrophin Is a Cdk5 Substrate That Restrains the Motility of Insulin Secretory Granules
Source: PLoS One. 2010 Sep 23;5(9):e12929. doi: 10.1371/journal.pone.0012929 (PMC2944849; doi:10.1371/journal.pone.0012929)
Supplement: Methods S1 — (0.04 MB DOC) [file pone.0012929.s001.doc]

*Supplement Methods*

**TIRFM:** Images were acquired with an Andor iXon+ DU897_BV (Andor Technology) EM-CCD camera on an Olympus IX 71 microscope using an Olympus PlanApo 100x/1.45NA TIRFM objective together with the 2x optovar of the microscope. The system was equipped with a 2-line Andor laser combiner [housing a Cobolt Calypso (491nm, 75mW) and a Cobolt Jive (561nm, 75mW; Cobolt AB) laser, an AOTF and an Andor PCU100 control unit]. The microscope was outfitted with a custom made TIRF condenser. Emission was detected through an OptoSplit II image splitter (Cairn Research Ltd.) equipped with a 565DCXD dichroic, a BrightLine HC 525/30, and a BrightLine HC 628/40 emission filter (Semrock). Automated image analysis was performed with the MotionTracking/Kalaimoscope software (Transinsight GmbH). The background of non-vesicular fluorescence was subtracted and the CgB-mRFP1^+^ granules were fitted by an analytical function as described [72]. The jitter of center definition was suppressed by smoothing the tracks with a sliding window (±1 frames). The speed was measured as a movement between every sequential frame. The upper accuracy of granule center definition estimated from the mean square displacement was 42nm and 30nm for GFP/CgB-mRFP1 and GFP-β2-syntrophin/CgB-mRFP1 cells, respectively.

**Electron microscopy.** Ultrathin sections were observed on a Tecnai12 Biotwin electron microscope (FEI Company) with a bottom mount TVIPS F214 CCD camera (Tietz Video and Image Processing Systems GmbH).

**Quantitative real-time PCR (qRT-PCR).** Following RT-PCR using an oligo d(T) primer, mRNA levels of rat β2-syntrophin and Cdk5 in INS-1 cells were measured by quantitative real-time PCR using the QuantiTect SYBR Green PCR Kit (Qiagen) and a MX4000 Thermocycler (Stratagene). Normalization was performed as previously described [78]. Primers for β2-syntrophin (β2-syn_s, β2-syn_as) and Cdk5 (Cdk5_s, Cdk5_as) are indicated in Supplement Table SVC.
