# Supplementary figures and images for: β2-Syntrophin Is a Cdk5 Substrate That Restrains the Motility of Insulin Secretory Granules
Source: PLoS One. 2010 Sep 23;5(9):e12929. doi: 10.1371/journal.pone.0012929 (PMC2944849; doi:10.1371/journal.pone.0012929)

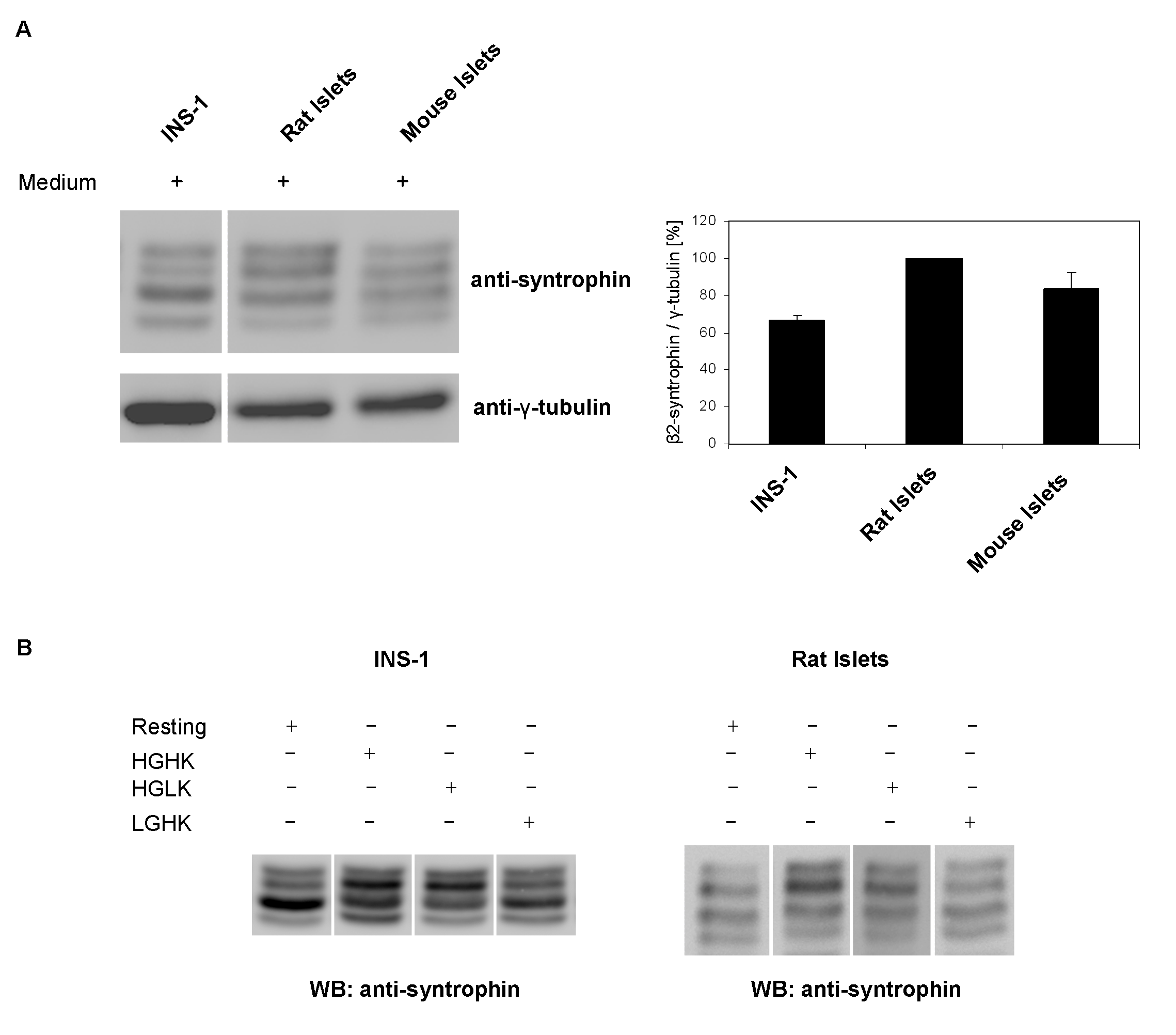

Supplement: Figure S1 — Expression pattern of β2-syntrophin in rat INS-1 cells and in rat and mouse islets. A) Immunoblotting (left panels) with anti-syntrophin and anti-γ-tubulin antibodies on extracts of INS-1 cells, rat and mouse islets kept in their standard culture medium with either 11 mM (INS-1 cells) or 5.5 mM (islets) glucose. Quantification (right histogram) of the immunoblots shown on the left. B) Immunoblotting with anti-syntrophin antibody on INS-1 cells (left panel) and rat islets (right panel). Cells were previously kept at rest (0 mM glucose, 5 mM KCl) or stimulated with high (25 mM) glucose and low (5 mM) KCl (HGLK), low (0 mM) glucose and high (55 mM) KCl (LGHK) or high glucose and high KCl (HGHK). (0.33 MB TIF) [file pone.0012929.s002.tif]

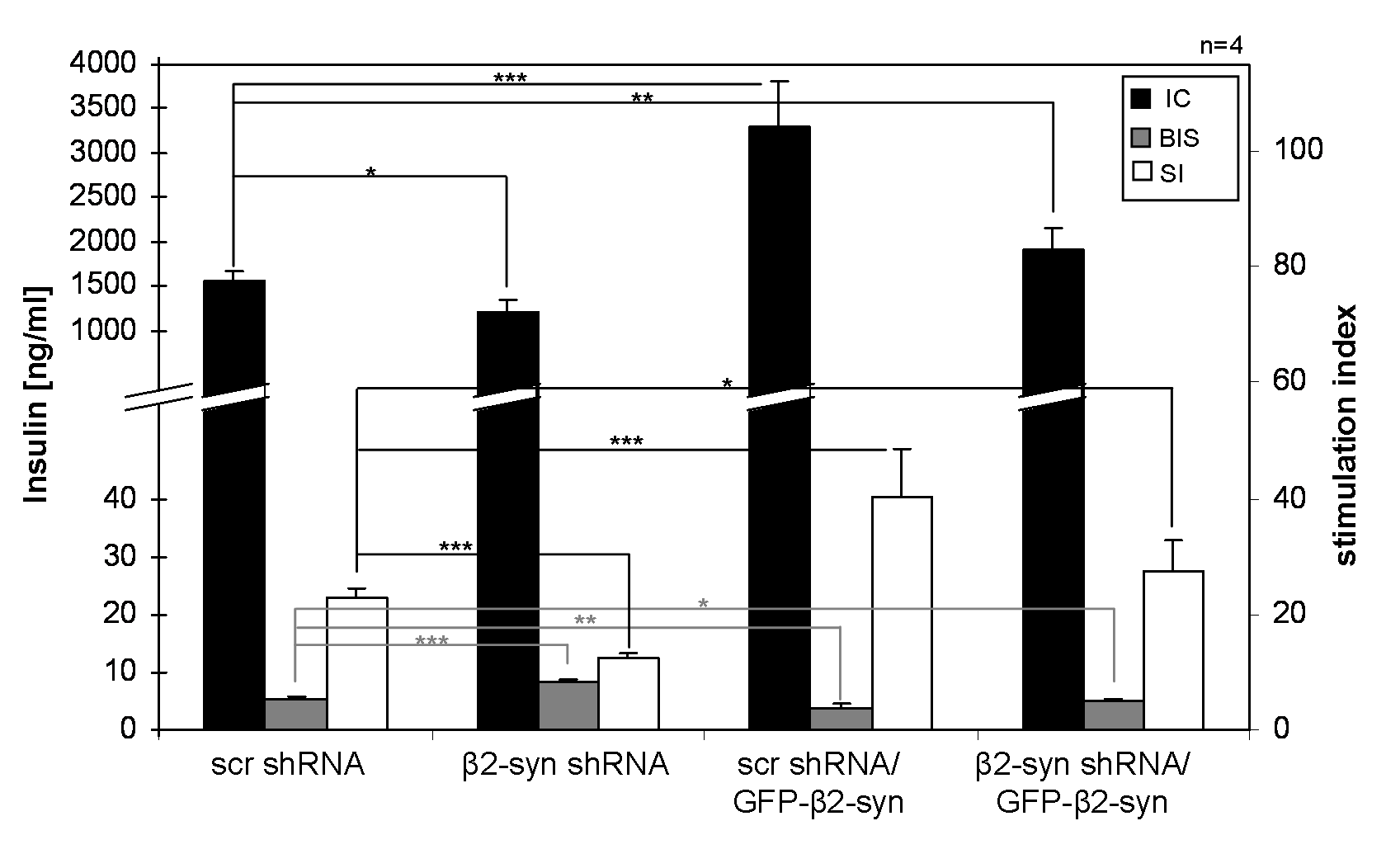

Supplement: Figure S2 — Impact of β2-syntrophin knockdown on insulin content and secretion. Insulin content (IC), basal insulin release (BIS) and insulin secretion Stimulation Index (SI) of INS-1 cells following the depletion of β2-syntrophin by RNA interference (β2-syn shRNA) and its rescue with GFP-β2-syntrophin. Control cells were transfected in parallel with a scrambled shRNA, with or without GFP-β2-syntrophin. n = number of independent experiments; *, p = 0.05; **, p = 0.01; ***, p = 0.005; p-values are relative to scr shRNA transfected INS-1 cells. (0.10 MB TIF) [file pone.0012929.s003.tif]

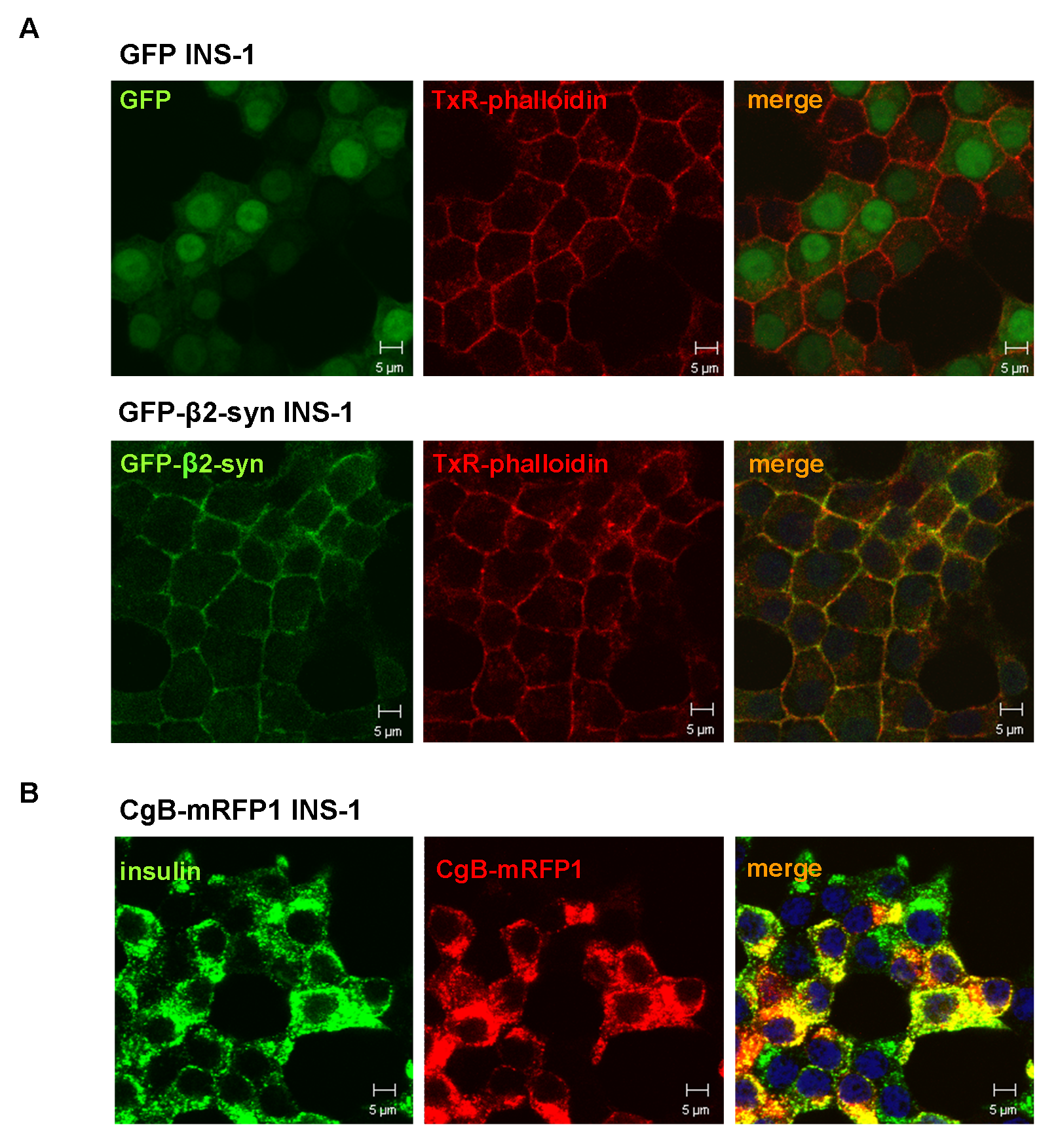

Supplement: Figure S3 — Colocalization of GFP-β2-syntrophin with F-actin and of CgB-mRFP1 with insulin. A) Confocal microscopy of GFP and GFP-β2-syntrophin INS-1 cells labeled with rhodaminated-phalloidin (pseudored). B) Co-localization of CgB-mRFP1 (pseudored) and insulin (pseudogreen) in stable CgB-mRFP1 INS-1 cells. Bars: 5 µm. (2.29 MB TIF) [file pone.0012929.s004.tif]

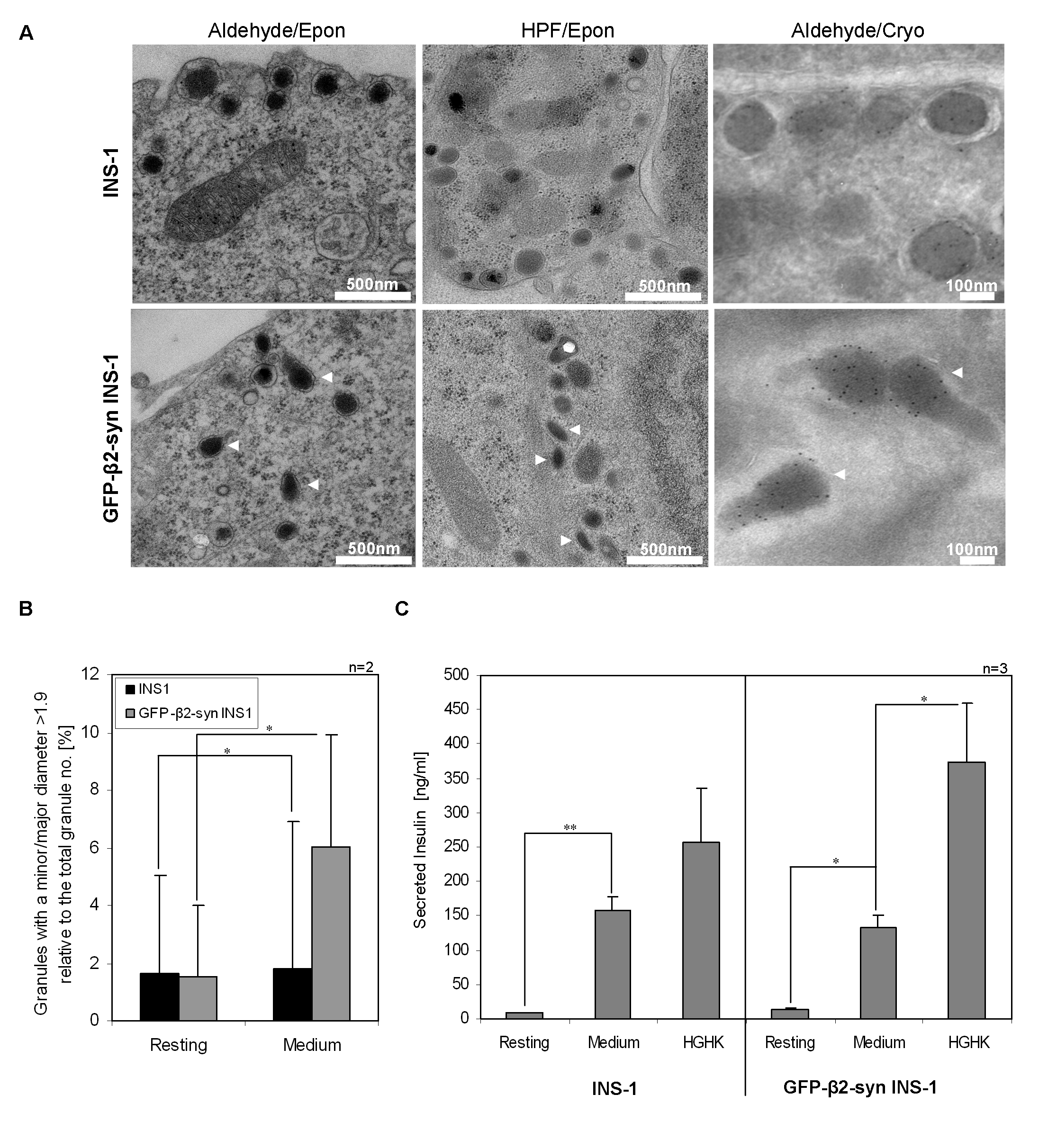

Supplement: Figure S4 — Granule morphometry in INS-1 cells and GFP-β2-syntrophin INS-1 cells. A) Electron microscopy images of INS-1 and GFP-β2-syntrophin INS-1 cells kept in culture medium and fixed with aldehydes (protocols 1 and 3) or by high-pressure freezing (HPF; protocol 2) followed by Epon-embedding (protocols 1 and 2) or freezing (Cryo; protocol 3) before sectioning. Aldehyde/cryo specimens were immunogold-labeled with an anti-insulin antibody. The black dots in the left panels correspond to immunogold-labeled insulin. White arrowheads point to granules with a deformed shape. B) Percentage of granules with a major/minor diameter ≥1.9 in resting or sub-maximally stimulated INS 1 and GFP-β2-syntrophin INS-1 cells. Data were obtained from 2 independent experiments (n). *, p = 0.05; C) Insulin secreted from INS-1 cells and GFP-β2-syntrophin INS-1 cells kept in culture medium, compared to resting and HGHK-stimulated cells. n = number of independent experiments; *, p = 0.05; **, p = 0.01. p-values in are relative to INS-1 cells and, GFP-β2-syntrophin INS 1 cells kept in culture media. (2.82 MB TIF) [file pone.0012929.s005.tif]

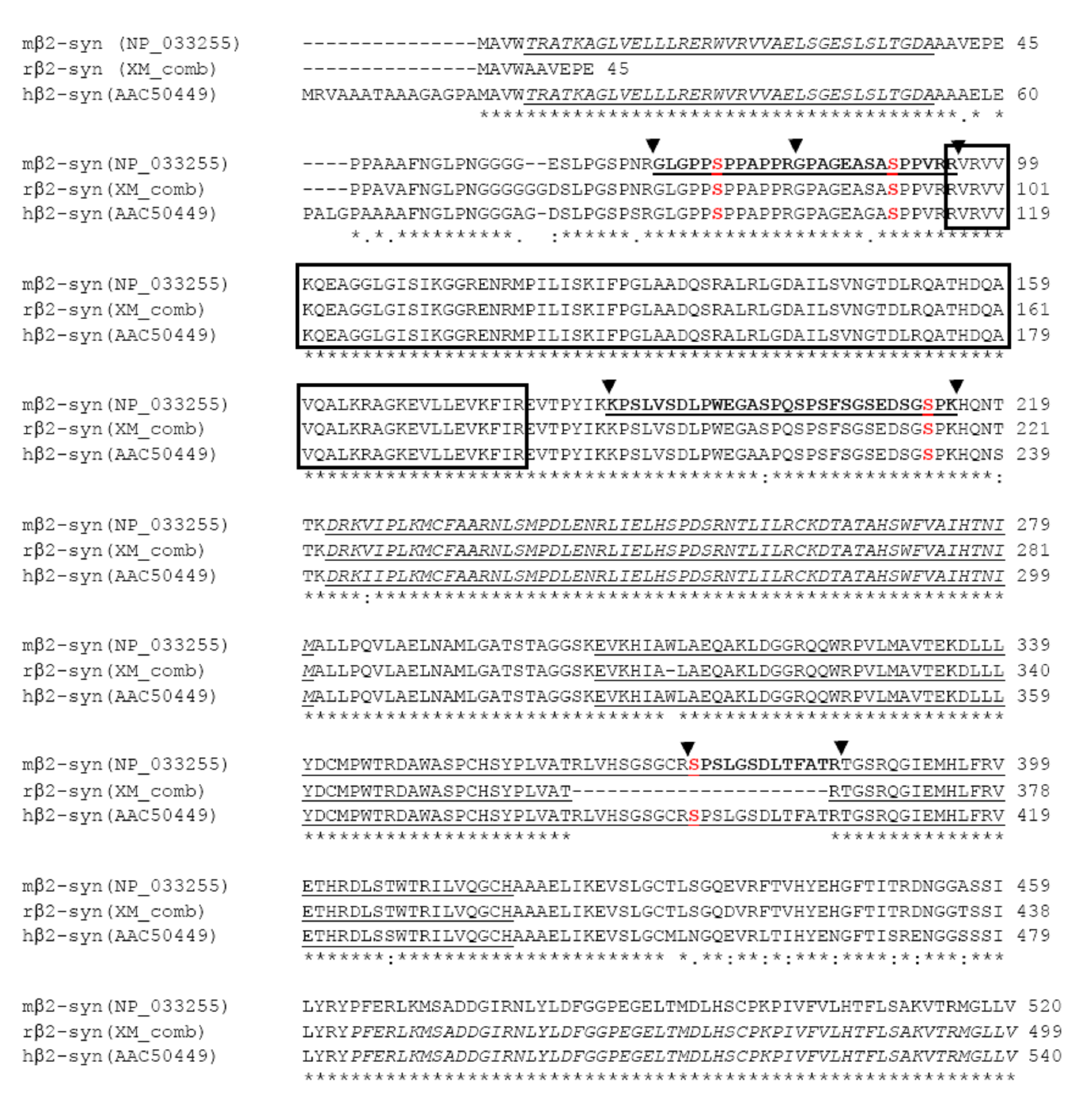

Supplement: Figure S5 — Conservation of phosphoserines in rodent and human β2-syntrophin. Alignment of the primary amino acid sequences of mouse (mβ2-syn), human (hβ2-syn) and predicted rat (rβ2-syn) β2-syntrophin. The split PH1 domain (PH1a, PH1b) of β2-syntrophin is marked in italic and underlined; the PH2 domain is underlined; the PDZ domain is boxed, while the SU domain is in italic. The phosphoserines of mouse β2-syntrophin identified by mass spectrometry are marked in red, while the isolated phosphopeptides are in bold and underlined. The rat β2-syntrophin sequence was assembled from two in silico sequences (XM_344764, XP_226430) of the rat genome project. Asterisks mark amino acids that are identical in mouse and human; double dots mark conservative changes, while single dots mark semi-conservative changes. (1.54 MB TIF) [file pone.0012929.s006.tif]

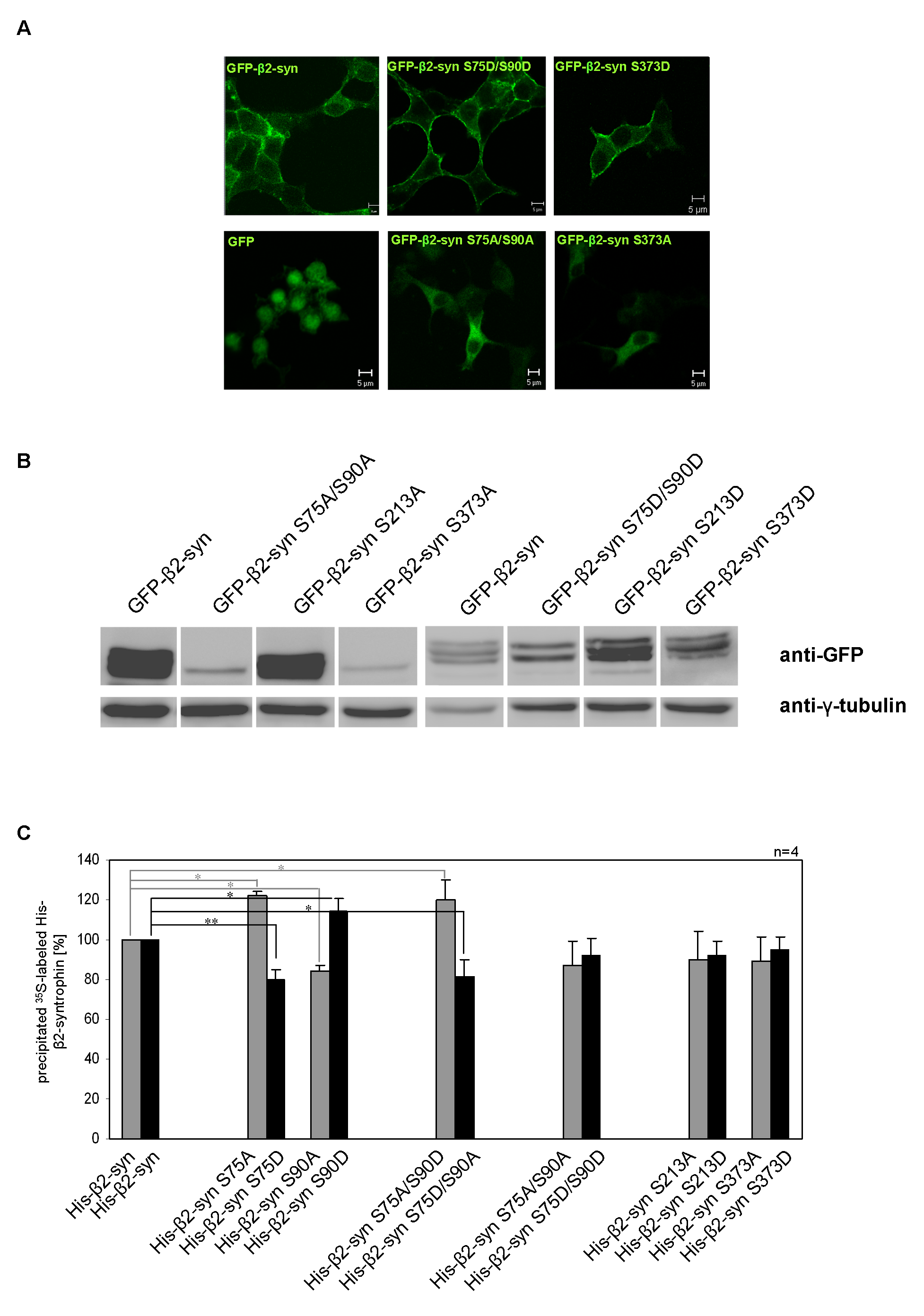

Supplement: Figure S6 — Localization, expression pattern and ICA512-binding of GFP-β2-syntrophin mutants. A) Confocal microscopy images of INS-1 cells expressing the indicated single or double GFP-β2-syntrophin S/D and S/A mutants compared to GFP-β2-syntrophin and GFP INS-1 cells. Bars: 5 µm. B) Immunoblots with anti-GFP and γ-tubulin antibodies on extracts of INS-1 cells expressing the GFP-β2-syntrophin variants. The left panel was overexposed relative to the right panel in order to detect GFP-β2-syntrophin S75A/S90A and S373A. C) Ratio of in vitro transcribed-translated 35S-His-β2-syntrophin phosphomutants pulled down with GST-ICA512601–979 relative to 35S-His-β2-syntrophin. n = number of independent experiments; *, p = 0.05; **, p = 0.01. (0.74 MB TIF) [file pone.0012929.s007.tif]

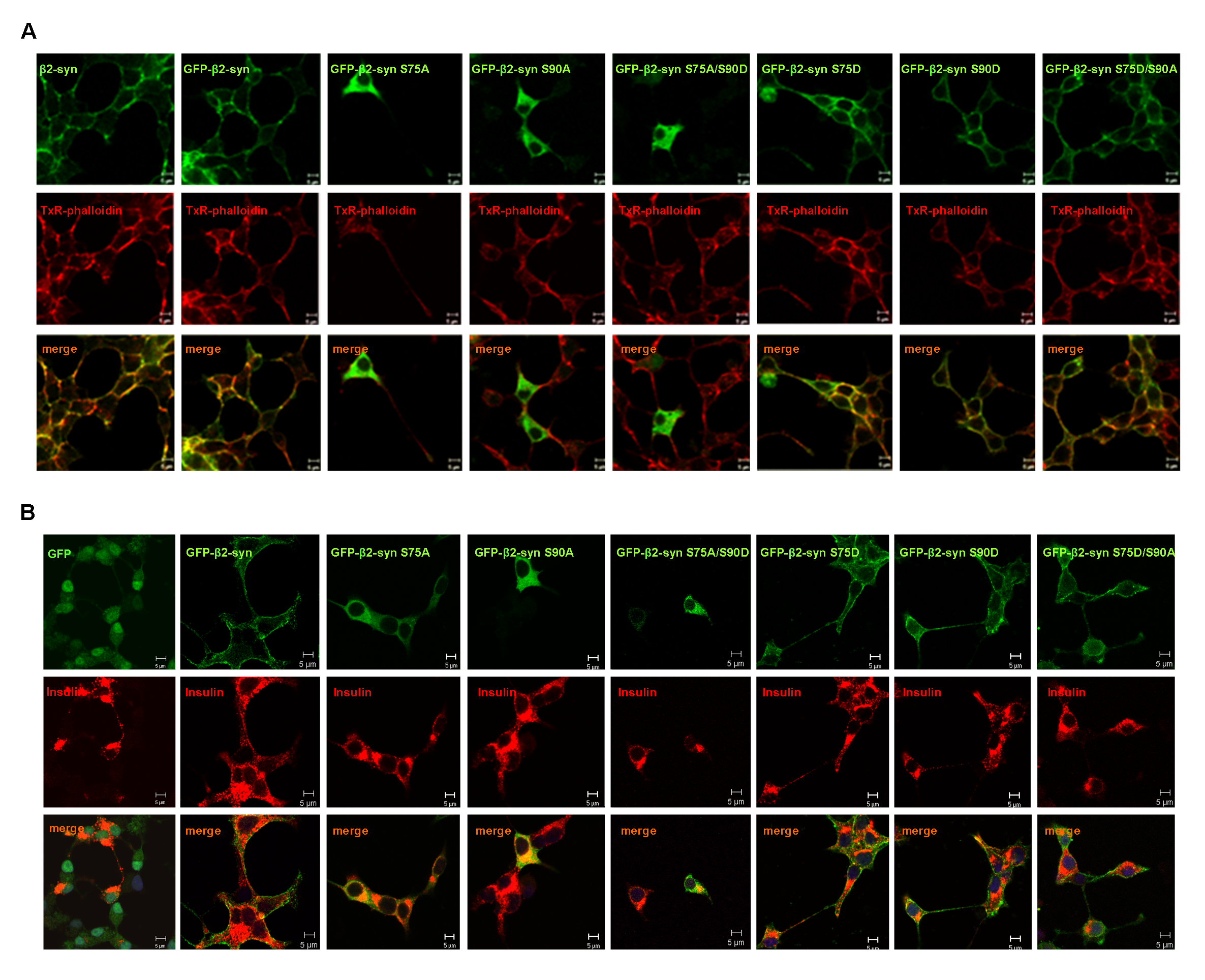

Supplement: Figure S7 — Colocalization of GFP-β2-syntrophin variants with the actin cytoskeleton and insulin. A) Colocalization of β2-syntrophin and GFP-β2-syntrophin variants with F-actin. B) Colocalization of β2-syntrophin and GFP-β2-syntrophin variants with insulin. Bars: 5 µm. (2.59 MB TIF) [file pone.0012929.s008.tif]

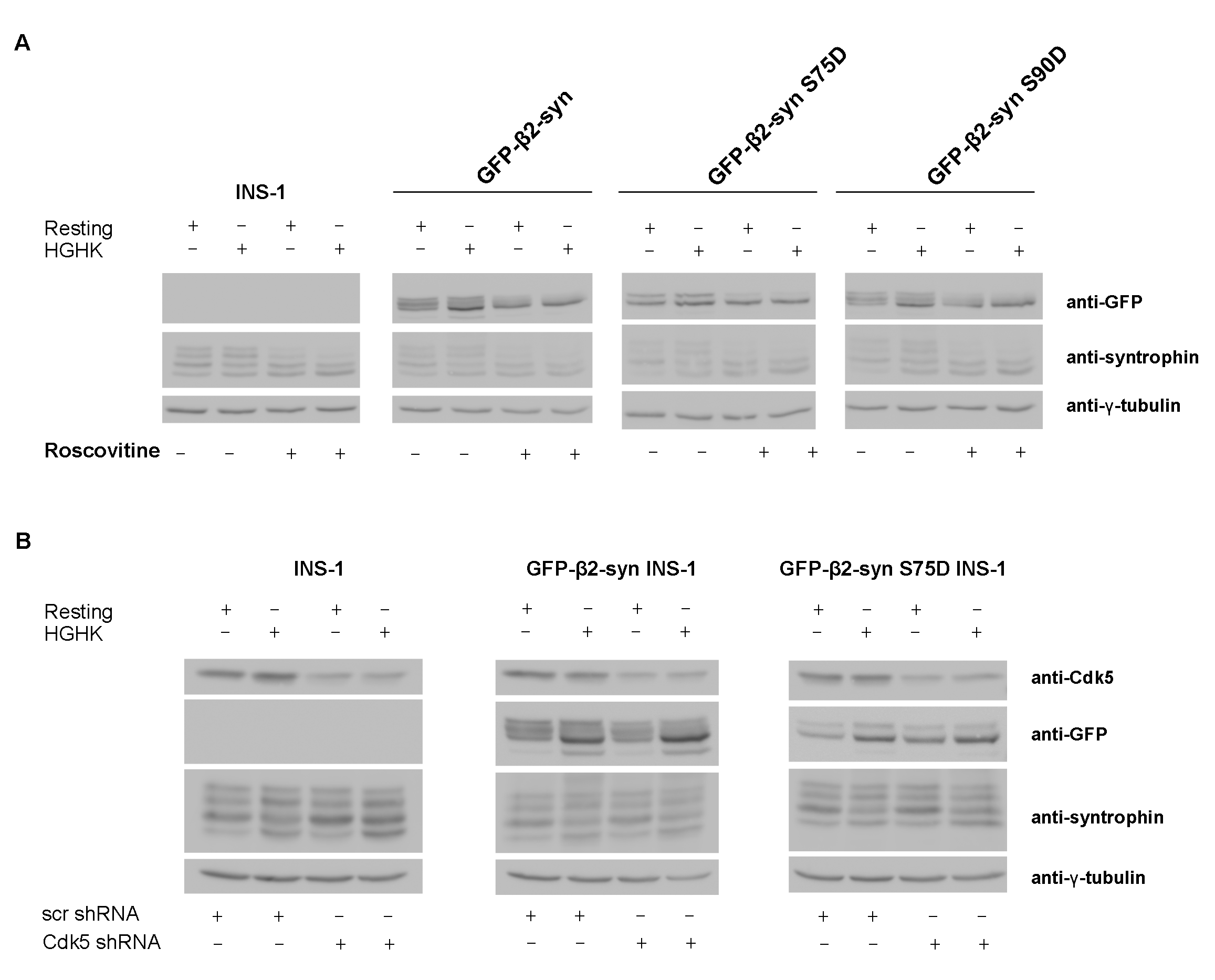

Supplement: Figure S8 — Impact of Cdk5 on the expression pattern of β2-syntrophin and GFP-β2-syntrophin variants in resting and stimulated INS-1 cells. A) Western blotting with anti-GFP, -syntrophin and -γ-tubulin antibodies on extracts from resting and HGHK-stimulated INS-1 cells (left panels) and INS-1 cells expressing different GFP-β2-syntrophin variants. Cells were treated or not with roscovitine prior to extraction. B) Immunoblots with anti-Cdk5, -GFP, -syntrophin, and -γ-tubulin antibodies on extracts of resting and HGHK-stimulated INS-1 (right panels), GFP-β2-syntrophin INS-1 (middle panels) and GFP-β2-syntrophin S75D INS-1 (left panels) cells following the knockdown of Cdk5 (Cdk5 shRNA). Control INS-1 cells were transfected with a scrambled shRNA. (0.42 MB TIF) [file pone.0012929.s009.tif]
