# Supplementary material for: β2-Syntrophin Is a Cdk5 Substrate That Restrains the Motility of Insulin Secretory Granules
Source: PLoS One. 2010 Sep 23;5(9):e12929. doi: 10.1371/journal.pone.0012929 (PMC2944849; doi:10.1371/journal.pone.0012929)
Supplement: Table S1 — Insulin content and secretion of INS-1 cells relative to GFP-β2-syntrophin expression and phosphorylation. A) Insulin content (IC), basal (BIS) and Stimulation Index (SI) of GFP-β2-syntrophin INS-1 cell clones G1, G6 and G8 compared to INS-1 cells and GFP INS-1 cells. B) IC, BIS and SI of INS-1 cells transfected with scr shRNA or β2-syn shRNA, with or without GFP-β2-syntrophin. C) IC, BIS and SI of INS-1 cells and INS-1 cells transiently transfected with GFP or GFP-β2-syntrophin variants. The data in A, B and C are from 3, 4 and 6 independent experiments, respectively. (1.07 MB PDF) [file pone.0012929.s010.pdf]

A) Beta2-syntrophin overexpression			
INS-1 cell clone	IC [ng/ml]	BIS [ng/ml]	SI
INS-1	1891 ± 16	3.95 ± 0.23 (100%)	42 ± 2.58 (100%)
G8	3083 ± 111 (p=0.018)	2.63 ± 0.04 (68%, p=0.001)	81 ± 2.45 (194%, p=0.001)
G6	2664 ± 195 (p=0.078)	3.31 ± 0.06 (83%, p=0.065)	60 ± 1.47 (145%, p=0.087)
G1	2359 ± 67 (p=0.038)	3.89 ± 0.12 (99%, p=0.453)	48 ± 3.31 (115%, p=0.017)
EGFP	1702 ± 137 (p=0.199)	4.8 ± 0.61 (129%, p=0.167)	39 ± 2.57 (95%, p=0.018)
**B) Beta2-syntrophin knockdown and rescue**			
transfected INS-1 cells	IC [ng/ml]	BIS [ng/ml]	SI
scr shRNA	1575 ± 99	5.54 ± 0.40 (100%)	23 ± 1.82 (100%)
β2-syn shRNA	1222 ± 127 (p=0.039)	8.5 ± 0.26 (154%, p=0.012)	13 ± 0.91 (56%, p=0.006)
GFP-β2-syn / scr shRNA	3291 ± 515 (p=0.018)	3.91 ± 0.24 (p=0.001)	40 ± 8.65 (p=0.009)
GFP-β2-syn / β2-syn shRNA	1906 ± 243 (p=0.014)	4.84 ± 0.44 (p=0.045)	28 ± 5.18 (p=0.002)
**C) Beta2-syntrophin phosphomutants**			
transfected INS-1 cells	IC [ng/ml]	BIS [ngml]	SI
INS-1	1038 ± 10.7	2.02 ± 0.08 (100%)	55 ± 3.33 (100%)
EGFP	1057 ± 18.1 (p=0.856)	1.9 ± 0.35 (94%, p=0.503)	54 ± 2.61 (98%, p=0.634)
GFP-β2-syn	1420 ± 4.9 (p=0.002)	1.2 ± 0.10 (61%, $p=1.5*10^{-7}$)	81 ± 12.94 (148%, p=0.005)
GFP-β2-syn S75A	1546 ± 12.4 (p=0.001)	1.8 ± 0.18 (89%, p=0.126)	63 ± 8.43 (115%, p=0.093)
GFP-β2-syn S90A	848 ± 14.5 (p=0.085)	2.4 ± 0.24 (120%, p=0.011)	43 ± 4.01 (78%, p=0.0005)
GFP-β2-syn S75D	736 ± 17.1 (p=0.030)	2.9 ± 0.58 (143%, p=0.020)	38 ± 4.21 (69%, p=0.0002)
GFP-β2-syn S90D	1716 ± 21.2 (p=0.003)	1.8 ± 0.09 (92%, p=0.105)	60 ± 9.63 (111%, p=0.249)
GFP-β2-syn S75A/S90D	1847 ± 19.0 (p=0.001)	1.4 ± 0.09 (71%, $p=8.08*10^{-7}$)	73 ± 13.27 (134%, p=0.035)
GFP-β2-syn S75D/S90A	580 ± 24.2 (p=0.024)	2.5 ± 0.2 (126%, p=0.02)	41 ± 1.39 (74%, p=0.022)
GFP-β2-syn S75A/S90A	1119 ± 9.7 (p=0.303)	2.0 ± 0.66 (100%, p=0.994)	55 ± 1.60 (102%, p=0.651)
GFP-β2-syn S75D/S90D	1313 ± 22.2 (p=0.084)	1.9 ± 0.09 (92%, p=0.567)	50 ± 4.62 (92%, p=0.138)
