# Supplementary material for: β2-Syntrophin Is a Cdk5 Substrate That Restrains the Motility of Insulin Secretory Granules
Source: PLoS One. 2010 Sep 23;5(9):e12929. doi: 10.1371/journal.pone.0012929 (PMC2944849; doi:10.1371/journal.pone.0012929)
Supplement: Table S2 — Granule density and mobility in GFP-β2-syntrophin INS-1 cells. A) Density of granules/100 µm2. B) Granule mean speed distribution; C) Mean square displacement presented as diffusion coefficient. The data are from 4 and 6 independent experiments, respectively. (0.97 MB PDF) [file pone.0012929.s011.pdf]

A) Granule density					
**Stimulus**	**CgB-mRFP INS-1 cells**	**SG/100µm^2**	**p-value**	**p-value**	no. of movies
**Resting**	**GFP**	**24 ± 3.10**			m=15
**HGHK**	**GFP**	**13 ± 2.62**	2.909E-10		m=15
**Resting**	**GFP-β2-syn**	**10 ± 4.39**		2.004E-11	m=17
**HGHK**	**GFP-β2-syn**	**6 ± 1.53**	1.767E-03	4.666E-09	m=12
**Resting**	**GFP**	**17.5 ± 4.76**		1.370E-16	m=64
**Resting**	**GFP-β2-syn**	**10.3 ± 2.97**	1.370E-16		m=49
**Resting**	**GFP-β2-syn S75D**	**15.2 ± 4.76**	0.01423	1.650E-10	m=64
**Resting**	**GFP-β2-syn S90D**	**12.1 ± 3.45**	9.314E-08	0.0057	m=32

B) Granule mobility - speed distibution					
**Stimulus**	**CgB-mRFP INS-1 cells**	**Speed Distrib. [µm/s]**	**p-value**	**p-value**	no. of movies
**Resting**	**GFP**	**0.455 ± 0.0697**			m=15
**HGHK**	**GFP**	**0.450 ± 0.0237**	0.8348		m=5
**Resting**	**GFP-β2-syn**	**0.338 ± 0.0254**		1.52E-05	m=17
**HGHK**	**GFP-β2-syn**	**0.403 ± 0.0152**	1.85E-07	0.0121	m=8
**Resting**	**GFP**	**0.516 ± 0.1434**		2.590E-09	m=64
**Resting**	**GFP-β2-syn**	**0.376± 0.0504**	2.590E-09		m=49
**Resting**	**GFP-β2-syn S75D**	**0.444 ± 0.1165**	0.00081	1.700E-04	m=64
**Resting**	**GFP-β2-syn S90D**	**0.380 ± 0.0465**	1.180E-06	0.6736	m=32

C) Granule mobility - Diffusion Coefficient					
**Stimulus**	**CgB-mRFP INS-1 cells**	**Diffusion Coeff. D [µm^2/s]**	**p-value**	**p-value**	no. of movies
**Resting**	**GFP**	**0.023 ± 0.004**			m=15
**HGHK**	**GFP**	**0.043 ± 0.009**	0.0097		m=5
**Resting**	**GFP-β2-syn**	**0.015 ± 0.003**		9.49E-05	m=17
**HGHK**	**GFP-β2-syn**	**0.047 ± 0.008**	0.0079	0.7540	m=8
**Resting**	**GFP**	**0.0189 ± 0.005**		2.23E-13	m=64
**Resting**	**GFP-β2-syn**	**0.0115 ± 0.003**	2.23E-13		m=49
**Resting**	**GFP-β2-syn S75D**	**0.0145 ± 0.006**	3.260E-05	0.0019	m=64
**Resting**	**GFP-β2-syn S90D**	**0.0128 ± 0.003**	5.120E-08	0.426	m=32
