# Supplementary material for: β2-Syntrophin Is a Cdk5 Substrate That Restrains the Motility of Insulin Secretory Granules
Source: PLoS One. 2010 Sep 23;5(9):e12929. doi: 10.1371/journal.pone.0012929 (PMC2944849; doi:10.1371/journal.pone.0012929)
Supplement: Table S3 — Granule morphometry in INS-1 cells and GFP-β2-syntrophin INS-1 cells. Total number of measured granules, number and percent of granules with a major/minor granule diameter ≥1.9 from INS-1 and GFP-β2-syn INS-1 cells in resting (R) buffer or in culture media (M). (0.21 MB PDF) [file pone.0012929.s012.pdf]

sample	total no. of Granules	major/minor diameter ≥1.9 [granule no.]	major/minor diameter ≥1.9 [%]	p-value	p-value	no. of images
**R INS-1**	**351 ± 24**	**4**	**1.63 ± 2.49**			9
**R GFP-β2-syn INS-1**	**240 ± 26**	**2**	**1.51 ± 3.39**	0.9396		7
**M INS-1**	**230 ± 10**	**5**	**1.81 ± 5.09**		0.9225	16
**M GFP-β2-syn INS-1**	**323 ± 19**	**23**	**6.04 ± 3.86**	0.0238	0.0158	12
