# Supplementary material for: β2-Syntrophin Is a Cdk5 Substrate That Restrains the Motility of Insulin Secretory Granules
Source: PLoS One. 2010 Sep 23;5(9):e12929. doi: 10.1371/journal.pone.0012929 (PMC2944849; doi:10.1371/journal.pone.0012929)
Supplement: Table S4 — Quantification of β2-syntrophin-ICA512 pull-down assays. Autoradiographic signals of in vitro transcribed-translated 35S-labeled His-β2-syntrophin S/D and S/A mutants recovered with GST-ICA512601–979 expressed as percentage of pull down His-β2-syntrophin. (0.33 MB PDF) [file pone.0012929.s013.pdf]

in vitro transcribed-translated products	^{35}S-labeled His-β2-syn [%]			
	SA	p-value	SD	p-value
His-β2-syn	100		100	
His-β2-syn 75	122 ± 2.00	p=0.0289	80 ± 5.15	p=0.0034
His-β2-syn 90	85 ± 2.50	p=0.0509	114 ± 6.98	p=0.0525
His-β2-syn S75A/S90D	120 ± 9.90	p=0.0519		
His-β2-syn S75D/S90A			82 ± 8.18	p=0.0434
His-β2-syn S75A/S90A	87 ± 12.63	p=0.4919		
His-β2-syn S75D/S90D			92 ± 8.4942	p=0.5206
His-β2-syn 213	90 ± 14.47	p=0.1587	92 ± 7.07	p=0.1254
His-β2-syn 375	89 ± 12.75	p=0.1734	95 ± 6.80	p=0.1366
