# Supplementary material for: β2-Syntrophin Is a Cdk5 Substrate That Restrains the Motility of Insulin Secretory Granules
Source: PLoS One. 2010 Sep 23;5(9):e12929. doi: 10.1371/journal.pone.0012929 (PMC2944849; doi:10.1371/journal.pone.0012929)
Supplement: Table S5 — Primers for A) site-chain-mutagenesis, B) RNAi and C) qRT-PCR. (0.69 MB PDF) [file pone.0012929.s014.pdf]

| | |
|---|---|
| **A) Oligonucleotides for Site-Chain-Mutagenesis** | |
| **S75A** | 5'-GCCTGGGGCCGCCTGCTCCTCCCGCGCCGCC-3' |
| **S90A** | 5'-GGAAGCGAGCGCCGCTCCTCCCGTGCGCCGAGTGC-3' |
| **S213A** | 5'-CTGGATGTCGGGGCCCCCTCCCTTGG-3' |
| **S373A** | 5'-CTGGATGTCGGGCCCCCTCCCTTGG-3' |
| **S75D** | 5'-GGCCTGGGGCCGCCGGATCCTCCCGCGCCGCCCC-3' |
| **S90D** | 5'-GGGGGAAGCGAGCGCGGATCCGCCCGTGCGCCGAG-3' |
| **S213D** | 5'-CAGGTTCTGGATGTCGGGGATCCCTCCCTTGGATCTGACC-3' |
| **S373D** | 5'-CAGGTTCTGGATGTCGGGATCCCTCCCTTGGATCTGACC-3' |
| **B) Oligonucleotides for RNA interference** | |
| **β2-syn shRNA** | 5'-TCTCGTAGCTA TCCACACCAACATATTCAAGAGATATGTTGGTGTGGATAGCTACCT-3' |
| **Cdk5 shRNA** | 5'-TCTCGCGCCATCATTCGAAACAATTCAAGAGATTGTTTCGAATGATGGCGCCT-3' |
| **scr shRNA** | 5'- TCTCGTGAAATAGAGTGTAGGAATTCAAGAGATTCCTACACTCTATTTCACCT-3´ |
| **C) Oligonucleotides for qRT-PCR** | |
| **β2-syn_s** | 5'-AACGAGT TCTGGAGCGCTCTC-3' |
| **β2-syn_as** | 5'-CTTCAGAATGATCTGGTAGAGG-3' |
| **Cdk5_s** | 5'-AACGAGTTCTGGAGCGCTCTC-3' |
| **Cdk5_as** | 5'-CTTCAGAATGATCTGGTAGAGG-3' |

β2-syn = β2-syntrophin; scr = scrambled, s = sense; as = antisense
